# Circular CPM promotes chemoresistance of gastric cancer via activating PRKAA2‐mediated autophagy

**DOI:** 10.1002/ctm2.708

**Published:** 2022-01-24

**Authors:** Lang Fang, Jialun Lv, Zhe Xuan, Bowen Li, Zheng Li, Zhongyuan He, Fengyuan Li, Jianghao Xu, Sen Wang, Yiwen Xia, Tianlu Jiang, Lu Zhang, Linjun Wang, Diancai Zhang, Hao Xu, Li Yang, Zekuan Xu, Weizhi Wang

**Affiliations:** ^1^ Division of Gastric Surgery, Department of General Surgery The First Affiliated Hospital of Nanjing Medical University Nanjing China; ^2^ Jiangsu Key Lab of Cancer Biomarkers Prevention and Treatment Collaborative Innovation Center for Cancer Personalized Medicine Nanjing Medical University Nanjing China

**Keywords:** 5‐FU resistance, autophagy, ceRNA, circRNA, gastric cancer, PRKAA2

## Abstract

**Background:**

Chemotherapy can significantly improve the disease‐free survival and overall survival of patients with advanced gastric cancer (GC). 5‐fluorouracil (5‐FU) is frequently applied in the clinic, acting as a first‐line chemotherapy drug of advanced GC, which could be used alone or combining platinum drugs. However, its efficacy is significantly attenuated by chemoresistance, which is associated with patients’ poor survival. Recently, there is evidence suggesting that dysregulation of autophagy may contribute to drug resistance in cancer, and circular RNAs (circRNAs) also take part in chemoresistance. However, whether circRNAs participate in 5‐FU chemoresistance through autophagy remains largely unknown.

**Methods:**

RNA sequencing technologies and bioinformatics analysis were performed in GC. Sanger sequencing, Actinomycin D assay and RNase R assay confirmed the circular structure of circular CPM (circCPM). Various cell line models and animal models were used to explore related functions in vitro and in vivo. Quantitative Real‐time PCR (qRT‐PCR), fluorescence in situ hybridization, ribonucleic acid; (RNA) pulldown assays, RNA binding protein immunoprecipitation assays and Luciferase reporter assays were applied to explore involved pathways.

**Results:**

circCPM was up‐regulated in 5‐FU resistant GC cell lines and tissue. Moreover, high circCPM expression is positively associated with poor survival. Silencing circCPM greatly improved chemosensitivity in vitro and in vivo. Mechanistically, it directly binds to miR‐21‐3p in the cytoplasm and therefore increases the expression of PRKAA2, contributing to the activation of autophagy and chemoresistance.

**Conclusion:**

Our results reveal that circCPM has a crucial role in regulating GC autophagy and 5‐FU resistance by targeting PRKAA2. It may function as a new theory basis for assessing the curative effect of GC and reversing 5‐FU chemoresistance.

## Graphical abstract

CircCPM is up‐regulated in 5‐FU resistant gastric cancer cells and tissues.

CircCPM modulates autophagy by working as a sponge of miR‐21‐3p, thereby up‐regulating PRKAA2 expression.

CircCPM regulates gastric cancer 5‐FU chemoresistance through the miR‐21‐3p/ PRKAA2 axis.

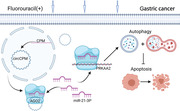

**Abbreviations**
5‐FU: 5‐fluorouracil
AMPK: AMP‐activated protein kinase
AV: autophagic vacuoles
c‐caspase3: cleaved caspase 3
circCPM: circular CPM
circRNA: circular RNA
CQ: chloroquine
FACS: fluorescence activated cell sorting
FISH: fluorescence in situ hybridization
GC: gastric cancer
LC3: microtubule‐associated protein 1 light chain 3
miRNA: microRNA
OS: overall survival
p62: sequestosome 1
PRKAA2: protein kinase AMP‐activated catalytic subunit alpha 2
TEM: transmission electron microscopy

## 1 INTRODUCTION

Gastric cancer (GC) is one of the most common malignant tumours with high fatality worldwide.^1^, ^2^ Its incidence is very high in Eastern Asia,^3^, ^4^ especially in China.^5^, ^6^ Despite the advances in early diagnosis and clinical treatment, the patients’ prognosis of with advanced GC is still poor, which has a low 5‐year overall survival (OS).^7^ Currently, chemotherapy based on 5‐FU and cisplatin is recommended for advanced GC patients.^8^, ^9^, ^10^

5‐fluorouracil (5‐FU) is one of the most widely used anti‐tumour agents, which shows significantly inhibitory effects against plenty of solid tumours.^11^, ^12^, ^13^ As a thoracic nucleotide synthase inhibitor, 5‐FU can interfere with deoxyribonucleic acid (DNA) and protein synthesis. There are studies showing that 5‐FU resistance seriously affects the prognosis of GC patients.^14^, ^15^, ^16^ Our previous studies also showed that TFF1 rs3761376 AA had a positive correlation with a worse prognosis among patients receiving 5‐FU‐based chemotherapy after surgery.^17^ Thus, the underlying mechanisms of 5‐FU resistance in GC patients need further exploration.

Autophagy, acting as a resistance mechanism against chemotherapy, received a great deal of attention in recent years.^18^ It is a lysosome‐mediated intracellular degradation process for proteins and organelles to maintain homeostasis and protects the cell from stress conditions, including hypoxia, metabolic stress and therapeutic agents.^19^, ^20^ Our previous studies have identified that a series of small‐molecule altered the GC cells chemotherapy sensitivity by modulating autophagy. For example, our team revealed that overexpressing miR‐148a‐3p in CDDP‐resistant cells inhibits cytoprotective autophagy by suppressing RAB12 and mTOR1 activation.^21^ miR‐1265 regulates GC autophagy by modulating CAB39 expression and the AMPK‐mTOR signaling pathway.^22^ However, the precise function and mechanism of autophagy in GC 5‐FU resistance needs further investigation.

Circular RNAs are a new type of non‐coding RNAs, which have a highly conserved closed‐loop structure, produced from pre‐mRNA back‐splicing.^23^, ^24^ They are believed to be highly stable without 5′ cap and 3′ polyadenylated tail. Accumulating evidence reveals that circRNAs contribute to diverse biological processes in cancer, including drug resistance.^25^, ^26^, ^27^, ^28^ Our previous study showed that circ‐AKT3 enhances cisplatin resistance by upregulating PIK3R1 expression in GC.^29^ However, up to now, few studies have revealed the regulatory mechanism of circRNAs in 5‐FU resistance. The functions of circRNAs in GC 5‐FU resistance need further investigation.

In this study, a reliable ceRNA network based on 5‐FU resistance was first constructed by using circRNA and messenger RNA (mRNA) microarray. We then focused on the key networks related to autophagy. With the advantage of RNA sequencing technologies, bioinformatics analysis and validation in GC tissues, we identified a circular CPM (circCPM)‐miR‐21‐3p‐PRKAA2 axis related to autophagy, which had a regulatory function in the 5‐FU resistance of GC. Our study provides a theoretical foundation for personalization in GC management and reversing drug resistance.

## 2 MATERIALS AND METHODS

### 2.1 Patients and samples

In total, 102 GC specimens were collected from the First Affiliated Hospital of Nanjing Medical University. Two samples obtained from patients receiving treatment with standard 5‐FU‐based neoadjuvant chemotherapy were used for sequencing. One hundred samples were used to analyse the expression of candidate circRNAs and relations between circCPM expression levels and clinical outcomes after radical resection in patients undergoing 5‐FU‐based adjuvant chemotherapy. 5‐FU resistance group consisted of patients whose disease‐free survival was <2 years, and 5‐FU sensitivity group consisted of patients whose disease‐free survival was ≥2 years among those receiving 5‐FU‐based adjuvant chemotherapy. The samples were obtained in 2017–2020. We collect specimens based on standard procedures. Within 30 min after the specimen was isolated, the cancer tissue was cut into several tissue blocks with a diameter of about 0.5 cm, which was respectively put into the numbered cryostorage tubes and quickly put into liquid nitrogen for long‐term preservation. This study was approved by the medical ethics committee of our hospital.

### 2.2 Cell culture

The human GC 5‐FU sensitive cells AGS and HGC‐27 as well as their resistant cell lines (AGS‐5FU and HGC‐27‐5FU) were used in this study. 5‐FU resistant GC cell strains were developed through gradually increasing 5‐FU treatment. The original concentration of 5‐FU started from 1uM (1/5 of IC50 values of the 5‐FU sensitive cells). Cells will be cultured in medium without chemotherapy agents after 24 h. The cells were cultured in medium with increasing 5‐FU concentration (1.5‐ to 2‐fold) after cells became stable. The resistant cell strains were eventually established by gradually increasing 5‐FU concentrations for 6 months. Then, the two cell lines were maintained in a complete medium containing 5‐FU. HGC‐27‐5FUand HGC‐27 were maintained in RPMI 1640 medium, while the rest were cultured in F12K medium. The HEK‐293T cell line was cultured in DMEM. These mediums were added with 10% fetal bovine serum. All cells were cultured in a cell incubator at 37°C in a constant atmosphere of 5% CO2.

### 2.3 RNA extraction and qRT‐PCR

RNA extraction and quantitative real‐time were performed as reported previously.^21^ The sequences of primers are displayed in Table S1.

### 2.4 CircRNA miRNA and mRNA expression profiles

The sequencing procedures and bioinformatics analysis were provided by the Shanghai Biotechnology Corporation (China). In short, 5‐FU sensitive and resistant tissues and cells (AGS‐5FU/AGS and HGC‐27‐5FU/HGC‐27) were used for ceRNA chips. After a series of professional treatments, the samples were analysed using miRNA sequencing and circRNA and mRNA chips (Agilent human ceRNA 3.0 chip; Agilent, CA, USA).

### 2.5 RNase R treatment

Total RNA was added for 20 min at 37°C with RNase R treatment at 3 U/μg (WI, USA). After RNA was treated with RNase R, reverse transcription was then performed under the manufacturer's instructions.

### 2.6 Actinomycin D assay

Actinomycin D assay was performed as described previously.^29^

### 2.7 Fluorescence in situ hybridization

Cy3‐labelled circCPM and FITC‐labelled miR‐21‐3p probes were specially provided by Servicebio (Wuhan, China). The fluorescence in situ hybridization (FISH) assay was performed as reported previously.^29^

### 2.8 Plasmid, siRNA and lentiviral construction

Human circCPM overexpression vector and si‐circCPM were purchased by Genechem (Shanghai, China). miRNA mimics and inhibitors were procured by GenePharma (Suzhou, China). PRKAA2 plasmids were purchased from Gene‐Pharma. The transfection process was performed with Lipofectamine 3000 according to the product manuals. The lentivirus vectors containing sh‐circCPM, sh‐PRKAA2 and overexpressing circCPM were purchased by Genechem (Shanghai, China). The stably transfected cell lines were selected with puromycin. The detailed sequences are shown in Table S1.

### 2.9 IC50 values

IC50 was detected as reported previously.^21^

### 2.10 Apoptosis assay

Annexin V PE Apoptosis Kit (BD, USA) was used. Apoptosis assay was performed as reported previously.^21^

### 2.11 Colony formation assays

Colony formation assays were carried out as reported previously.^21^

### 2.12 GFP‐mRFP‐LC3 imaging

GFP‐mRFP‐LC3 imaging was conducted as reported previously.^21^ Puncta number was counted in six different microscope fields.

### 2.13 Transmission electron microscopy

Transmission electron microscopy (TEM) was conducted as reported previously.^21^ The number of autophagic vacuoles (AV) was counted in 15 different cells.

### 2.14 WB

Western blotting was performed as reported previously.^29^ Details of the antibodies are shown in the Table S2.

### 2.15 RNA pulldown assay and immunoprecipitation assay

A pulldown assay and RNA binding protein immunoprecipitation (RIP) assay were performed as reported previously.^29^

### 2.16 Luciferase assay

Luciferase reporter vectors containing the wild‐type fragments of circCPM or 3′‐UTR of PRKAA2 were constructed by RiboBio (Guangzhou, China). Luciferase assay was performed as reported previously.^29^

### 2.17 Immunohistochemistry staining

Immunohistochemistry (IHC) was performed as reported previously.^29^

### 2.18 Organoid culture and viability assay

GC tissues were obtained from the department of gastric surgery in a sterile condition. Tissues were finely chopped and digested with collagenase A at 37°C for 40 min. Then, the cell suspension was mixed in Matrigel (R&D Systems, USA) supplemented with several growth factors added at a concentration of 100 ng/ml. Fifty microliters of Matrige per well were added to a 24‐well plate. After that, 500 μl of human organoid culture medium (Stemcell Technologies, Canada) was added to each well for organoid growth. Organoids were transfected with siRNAs using Lipofectamine 3000. Photographs were taken daily by microscope. Cell viability was analysed using the PrestoBlue Cell Viability Reagent (Invitrogen). For the chemotherapy using 5‐FU, organoids were overlaid with medium for 48 h. PrestoBlue reagent (1x) was added to organoids and incubated for 3 h at 37°C. Absorbance (Tecan Reader, Genio) was measured.

### 2.19 Animal study

Animal experiments were performed under the instructions of animal center in our university. Nude mouse xenograft model was performed as reported previously.^29^

### 2.20 Statistical analysis

SPSS 20.0 and GraphPad Prism 7.0 software were used to analyse the experimental data and clinical data, which included one‐way analysis of variance, student's *t* test, Kaplan–Meier analysis and logrank test.

## 3 RESULTS

### 3.1 Dysregulated circRNAs in 5‐FU resistant GC

**FIGURE 1:**
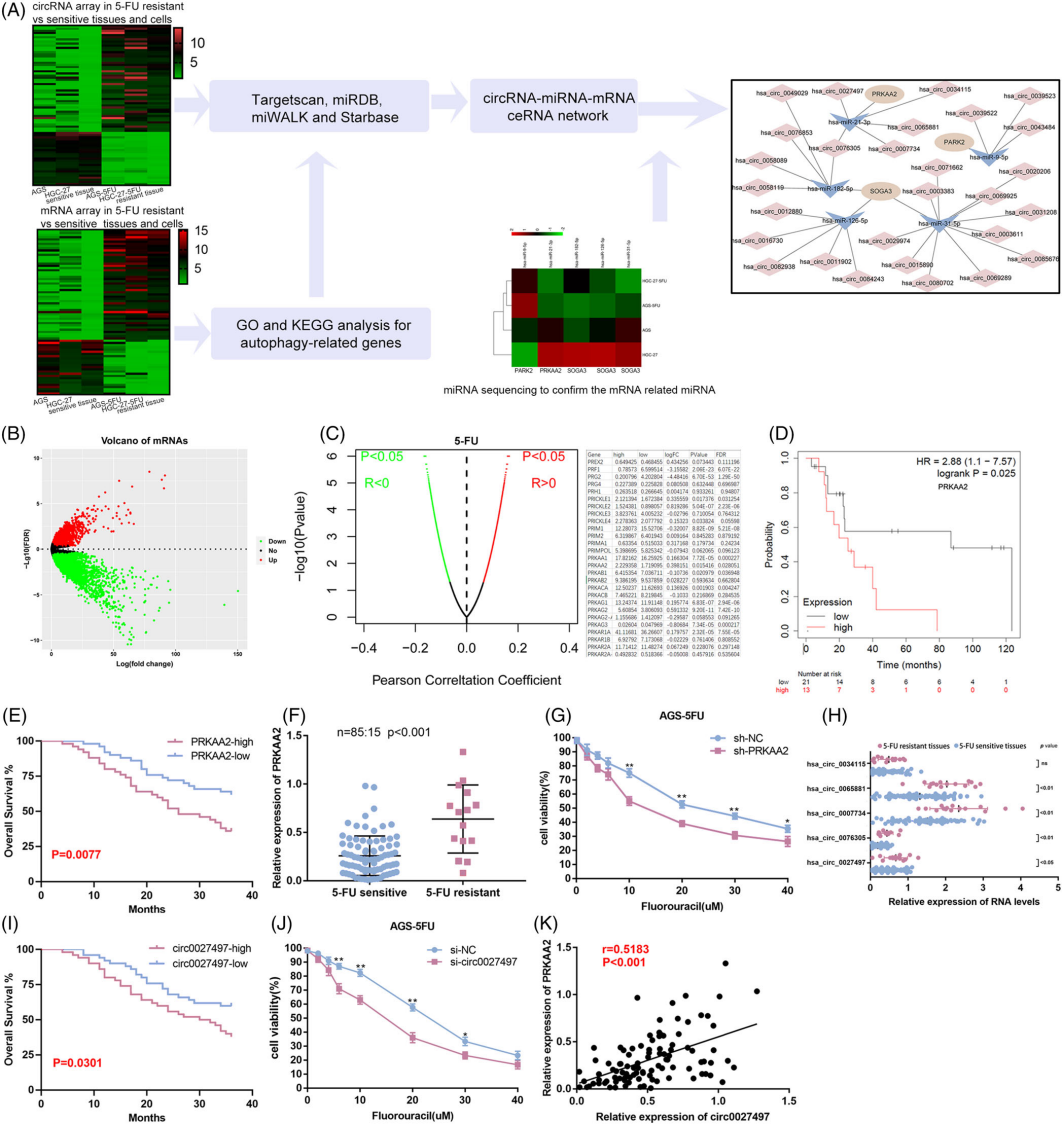
Dysregulated circRNAs in chemoresistant gastric cancer (GC). (A) Schematic illustration of screening the autophagy‐related ceRNA regulatory network in regulating 5‐fluorouracil (5‐FU) resistance. (B) Volcano plot for the mRNA matrix generated on the basis of genomics of drug sensitivity in cancer (GDSC) statistics. (C) Left panel: Pearson correlation between genes and 5‐FU sensitivity according to the GDSC database. Right panel: PRKAA2 was negatively correlated with the IC50 values for 5‐FU. (D) The Kaplan‐Meier (KM) plotter data of gastric cancer patients receiving 5‐FU‐based chemotherapy. (E) Kaplan–Meier analysis of the correlation between PRKAA2 expression and overall survival. (F) Expression of PRKAA2 in 5‐FU‐resistant and 5‐FU‐sensitive GC tissues by qRT‐PCR. (G) CCK8 assay of the effect of silencing PRKAA2 on the drug sensitivity of AGS‐5FU cells. (H) Expression of five‐candidate circRNAs in 5‐FU‐resistant and 5‐FU‐sensitive GC tissues by qRT‐PCR. (I) Kaplan–Meier analysis of the correlation between circ0027497 expression and overall survival. (J) CCK8 assay of the effect of silencing circ0027497 on the drug sensitivity of AGS‐5FU cells. (K) Pearson correlation analysis between the expression of circ0027497 and PRKAA2

To investigate the circRNA and mRNA expression profiles, we performed combined analysis by using a circRNA and mRNA microarray in 5‐FU resistant and sensitive GC cells and tissues (Figure 1A). GC cells include AGS/AGS‐5FU and HGC‐27/HGC‐27‐5FU. GC tissues were obtained from two patients receiving neoadjuvant chemotherapy based on 5‐FU. We found hundreds of up‐regulated or down‐regulated circRNAs and mRNAs in 5‐FU resistant GC cell lines and tissues. We first established a ceRNA regulatory network by Targetscan, miRDB, miWALK and Starbase databases (fold change (FC) > 2, FC < 0.5, *p* < 0.05). Considering the critical role of autophagy in drug resistance, we further analysed autophagy pathway‐related genes including PARK2, PRKAA2 and SOGA3 by clusterProfiler for GO and KEGG. Next, genomics of drug sensitivity in cancer (GDSC) database was used to analyse relation between these autophagy pathways‐related genes and 5‐FU sensitivity. Dividing genes into two groups according to the IC50 value of 5‐FU drug sensitivity (false discovery rate (FDR) < .05 FC > 1.3), we found that the expression of PRKAA2 was up‐regulated (Figure 1B). And analysis of the Pearson correlation coefficient between genes and 5‐FU drug sensitivity indicated that high PRKAA2 expression was positively correlated with 5‐FU resistance (Figure 1C). Kaplan–Meier plotter results displayed that only patient with high PRKAA2 expression had worse survival compared with those with low PRKAA2 expression (https://kmplot.com/analysis/) (Figure 1D). Follow‐up data from our center also had similar results (Figure 1E). qRT‐PCR analysis showed that PRKAA2 expression was up‐regulated in 5‐FU resistant tissues (Figure 1F). Cell viability analysis also confirmed that reduced PRKAA2 expression facilitated the chemosensitivity in chemoresistant cells (Figure 1G and Figure S1A). Based on the preliminary constructed ceRNA regulatory network, we performed miRNA second‐generation sequencing to optimize the ceRNA network further. Combing bioinformatic prediction and sequencing results, we found several mRNA‐related miRNAs, which included hsa‐miR‐21‐3p, hsa‐miR‐9‐5p, hsa‐miR‐162‐5p, hsa‐miR‐126‐5p and hsa‐miR‐31‐5p (FC > 2). Then, we selected five‐candidate circRNAs that may regulate PRKAA2. Results of qRT‐PCR showed that circ0027497 expression (also termed as circCPM in this study) was up‐regulated in 5‐FU resistant tissues (Figure 1H). Follow‐up data analysis showed only high circCPM expression had a negative correlation with patients’ survival (Figure 1I and Figure S1B‐E). Cell viability assays showed reduced circCPM expression reduced IC50 in 5‐FU resistant GC cells (Figure 1J and Figure S1F‐N). Linear correlation pattern analysis indicated a positive association between PRKAA2 expression and circCPM expression (Figure 1K). There is one miR‐21‐3p that links circCPM and PRKAA2 in the ceRNA regulatory network. Therefore, we ultimately chose the circCPM‐miR‐21‐3p‐PRKAA2 axis for subsequent studies.

### 3.2 Characterizations of circCPM

**FIGURE 2:**
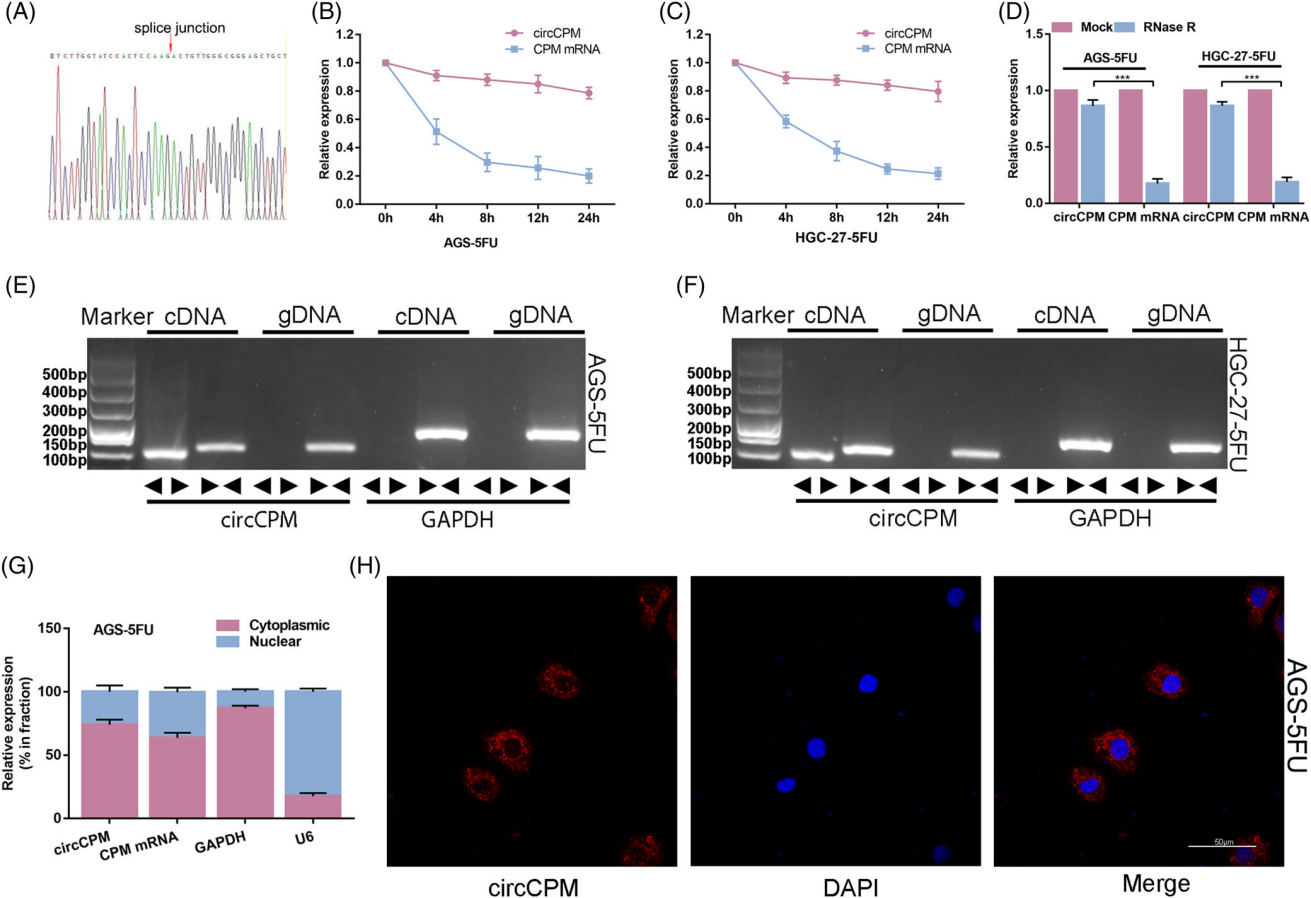
Characterization of circular CPM (circCPM). (A) Validation of head‐to‐tail splicing of circCPM using Sanger sequencing. (B and C) The relative expression changes of circCPM and CPM mRNA in AGS‐5FU and HGC‐27‐5FU after actinomycin D treatment for 4 h, 8 h, 12 h and 24 h. (D) The relative expression changes of circCPM and CPM mRNA in AGS‐5FU and HGC‐27‐5FU after RNase R treatment. (E and F) RT‐PCR‐based detection of circular and linear CPM using convergent and divergent primers in cDNA and genomic DNA (gDNA). (G) qRT‐PCR analysis confirming that circCPM and linear CPM are mainly located in the cytoplasm. (H) Fluorescence in situ hybridization (FISH) results depicting the cytoplasm location of circCPM. Scale bar = 5 μm. (Graph represents mean ± SD; *p < .05, **p < .01 and ***p < .001)

CircCPM originates from the fourth, fifth and sixth exons of Carboxypeptidase M (CPM) genes, which has not been described previously. Sanger sequencing identified the head‐to‐tail splicing structure of circCPM with expected size (Figure 2A). Actinomycin D assay showed circCPM expression was not affected while linear CPM mRNA expression decreased (Figure 2B,C). And circCPM was more resistant to RNase R compared with linear CPM mRNA (Figure 2D). Next, to test the circular structure of circCPM, we designed divergent primers and convergent primers to amplify circCPM and linear CPM mRNA. Complementary DNA (cDNA) and gDNA extracted from AGS‐5FU and HGC‐27‐5FU were applied as templates (Figure 2E,F). The results indicated that divergent primers only amplified circCPM in cDNA. The qRT‐PCR result demonstrated circCPM was predominantly localized within the cytoplasm (Figure 2G and Figure S1O). FISH results demonstrated similar results (Figure 2H). Collectively, our results implied that circCPM is a stable and cytoplasmic circular RNA derived from CPM, which may play an essential role in 5‐FU resistance.

### 3.3 CircCPM enhances GC 5‐FU chemoresistance and autophagy in vitro

To determine the biological functions of circCPM in GC chemoresistance, we constructed circCPM overexpressing cells and circCPM knockdown cells in 5‐FU sensitive and resistant cells, respectively (Figure S1P‐S).

**FIGURE 3:**
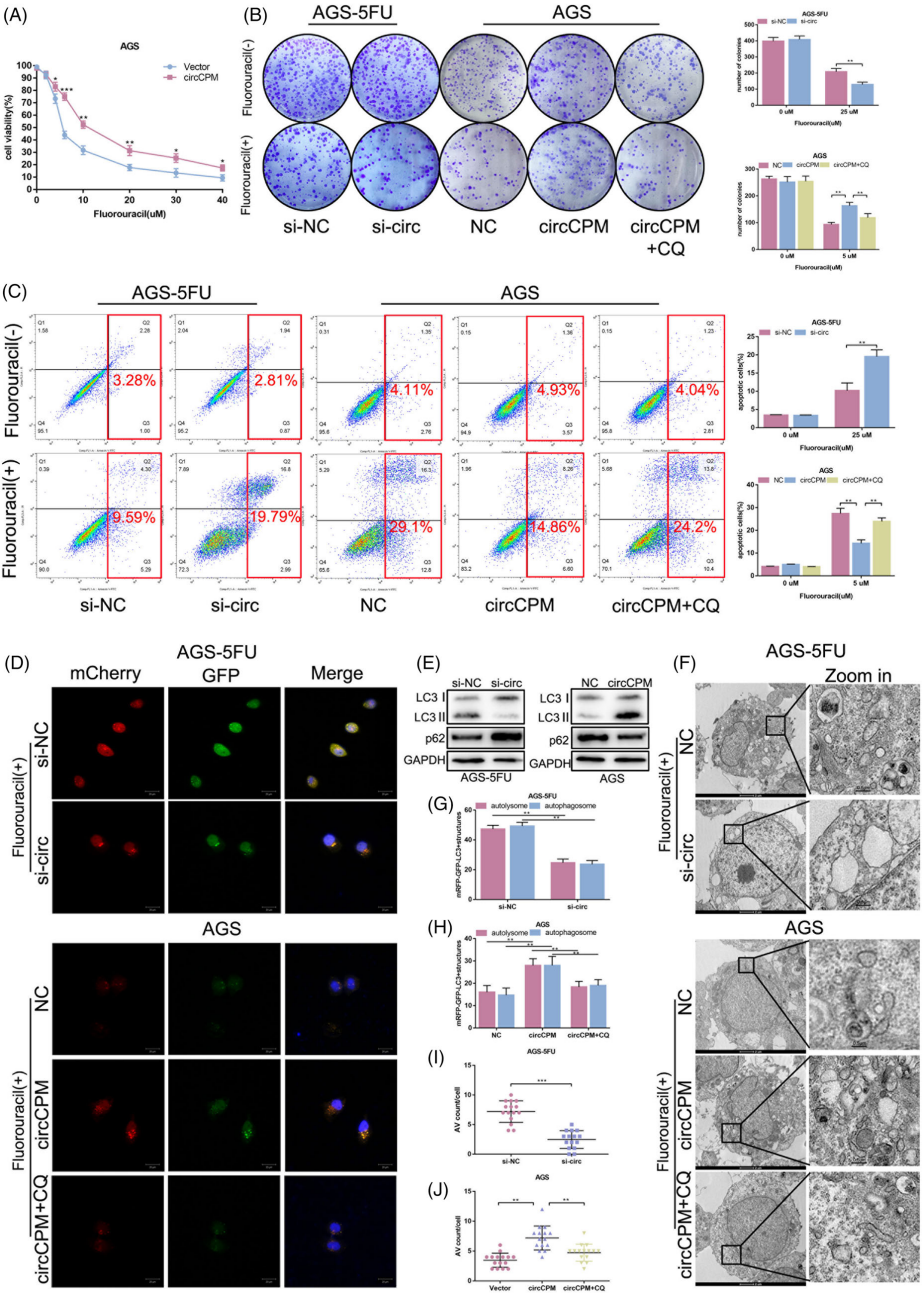
Circular CPM (CirCPM) enhances gastric cancer (GC) chemoresistance via autophagy in vitro. (A) The effect of overexpressing circCPM on the drug sensitivity of GC cells was measured by a CCK8 assay. (B) Colony formation assays of AGS‐5FU and AGS cells were performed to assess the proliferative ability. Right upper panel: Quantification data for AGS‐5FU transfected with si‐circ with or without 5‐FU exposure (25 μM 48 h). Right lower panel: Quantification data for AGS transfected with circCPM overexpression plasmids with or without 5‐FU (5 μM 48 h) and/or chloroquine (CQ) (20 μM 24 h) exposure. (C) Apoptotic assays of GC cells to assess circCPM modulation on the drug sensitivity. Right upper panel: Quantification data for AGS‐5FU transfected with si‐circ with or without 5‐FU exposure (25 μM 48 h). Right lower panel: Quantification data for AGS transfected with circCPM overexpression plasmids with or without 5‐FU (5 μM 48 h) and/or CQ (20 μM 24 h) exposure. (D, G and H) Immunofluorescence analysis using GFP‐mRFP‐LC3 staining. (G and H) The numbers of LC3 puncta (yellow puncta for autophagosome and red puncta for autolysosome) were quantified in AGS‐5FU transfected with si‐circ upon 5‐FU exposure (25 μM 48 h) and AGS transfected with circCPM overexpression plasmids upon 5‐FU (5 μM for 48 h) and/or CQ (20 μM 24 h) exposure. Scale bar 20 μm. (E) Western blot analysis of LC3 and p62 protein levels in cells transfected with si‐circ or circCPM overexpression vectors in AGS‐5FU and AGS. (F, I and J) Transmission electron microscopy (TEM) images of ultrastructure microstructure in representative AGS‐5FU and AGS cells transfected with si‐circ and circCPM overexpression plasmids. (I and J) The number of autophagic vacuoles (AV) of 15 cells was counted in each section. Scale bar = 2 μm or 0.5 μm. (Graph represents mean ± SD; *p < .05, **p < .01 and ***p < .001)

Subsequently, the results of cell viability showed that reduced circCPM expression facilitated the chemosensitivity in 5‐FU resistant GC cells, with a decrease in the IC50 value (Figure 1J and Figure S1J). However, overexpressing circCPM in 5‐FU sensitive GC led to the opposite results (Figure 3A and Figure S2A). Besides, plate colony formation assay and cell apoptosis were also examined. Chloroquine (CQ), an autophagy inhibitor, was also applied in these functional experiments. The results showed that reducing circCPM expression decreased the plate colony numbers and increased the apoptosis proportion in 5‐FU resistant cells, whereas enhancing circCPM expression in 5‐FU sensitive cells led to the opposite results (Figure 3B,C and Figure S2B,C). Then, we further explored the potential function of circCPM on autophagy. Silencing and overexpression of circCPM inhibited and promoted the basic autophagic level in 5‐FU resistant and sensitive cells as determined by LC3 and p62 levels (Figure 3E and Figure S2E). Furthermore, the numbers of LC3 dots increased after overexpressing circCPM in 5‐FU sensitive cells, while decreased after silencing circCPM in 5‐FU resistant cells (Figure 3D,G,H and Figure S2D,G,H). The results of TEM confirmed that silencing circCPM resulted in decreased AV counts. The opposite result was observed with exogenous expression of circCPM in 5‐FU sensitive cells (Figure 3F,I,J and Figure S2F,I,J).

### 3.4 CircCPM functions as a sponge of miR‐21‐3p in GC

**FIGURE 4:**
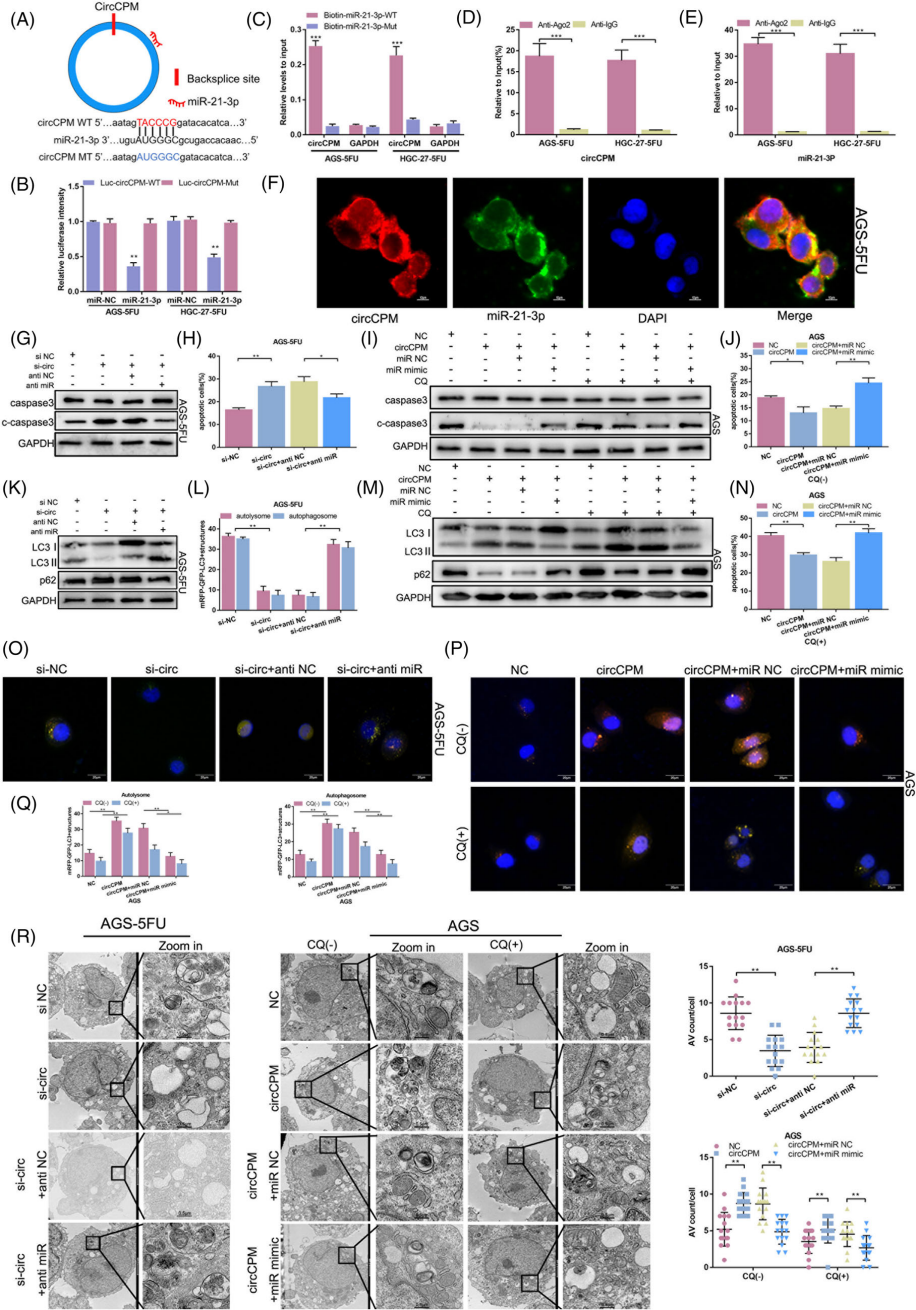
Circular CPM (CircCPM) functions as a sponge of miR‐21‐3p in gastric cancer (GC). (A) Schematic illustration of the predicted miR‐21‐3p binding sites on circCPM. (B) A luciferase reporter assay was used to detect the binding ability of circCPM and miR‐21‐3p in AGS‐5FU and HGC‐27‐5FU cell lines. (C) The biotinylated wild‐type/mutant miR‐21‐3p was transfected into AGS‐5FU and HGC‐27‐5FU cell lines with circCPM overexpression. The circCPM levels were examined by qRT‐PCR after capture. (D and E) RNA binding protein immunoprecipitation (RIP) assays were performed to assess the binding ability of miR‐21‐3p or circCPM and AGO2. (F) Fluorescence in situ hybridization (FISH) showed that circCPM and miR‐21‐3p were co‐localized in the cytoplasm in AGS‐5FU cells. The nucleus staining with 4,6‐diamino‐2‐phenyl indole (DAPI) (blue). (G and K) Western blot analysis of caspase3, c‐caspase 3, LC3 and p62 in AGS‐5FU transfected with si‐circ or co‐transfected with anti‐miR upon 5‐FU exposure (25 μM 48 h). (I and M) Western blot analysis of caspase3, c‐caspase 3, LC3 and p62 in AGS transfected with circCPM overexpression vector or co‐transfected with miR mimic upon 5‐FU exposure (5μM for 48h). (H) Apoptotic assays of AGS‐5FU cells transfected with si‐circ or co‐transfected anti‐miR upon 5‐FU exposure (25 μM 48 h). (J and N) Apoptotic assays of AGS cells transfected with circCPM overexpression vector or co‐transfected with miR mimic upon 5‐FU (5 μM 48 h) and/or chloroquine (CQ) (20 μM 24 h) exposure. (L and O) Immunofluorescence analysis of AGS‐5FU transfected with si‐circ or co‐transfected anti‐miR upon 5‐FU exposure (25 μM 48 h). Scale bar 20 μm. (L) Quantification data of autolysosome and autophagosome in AGS‐5FU. (P and Q) Immunofluorescence analysis of AGS transfected with si‐circ or co‐transfected anti‐miR upon 5‐FU (5 μM 48 h) and/or CQ (20 μM 24 h) exposure. Scale bar 20μm. (P) Quantification data of autolysosome and autophagosome in AGS. (R) Transmission electron microscopy (TEM) images of AGS‐5FU and AGS with specific treatments. Right upper panel: Quantification data of autophagic vacuoles (AV) counts in AGS‐5FU transfected with si‐circ or co‐transfected anti‐miR upon 5‐FU exposure (25 μM 48 h). Right lower panel: Quantification data of AV counts in AGS transfected with si‐circ or co‐transfected anti miR upon 5‐FU (5 μM 48 h) and/or CQ (20 μM 24 h) exposure. The number of AV of 15 cells was counted in each section. Scale bar = 2 μm or 0.5 μm. (Graph represents mean ± SD; *p < .05, **p < .01 and ***p < .001)

It is well known that circRNAs have a sponge‐like effect on miRNAs, and circCPM is mainly enriched in cytoplasm. Therefore, we investigated the probability of circCPM binding to miRNAs. CircCPM was predicted to potentially bind to miR‐21‐3p by TargetScan (Figure 4A). To directly confirm circCPM binds to miR‐21‐3p, we designed a wild‐type and mutant luciferase plasmid based on the predicted binding sites. The luciferase reporter assay results displayed miR‐21‐3p extremely reduced luciferase activity in 293T cells transfected with Luc‐circCPM‐WT plasmid (Figure 4B). A miRNA pulldown assay revealed that biotinylated miR‐21‐3p greatly enriched circCPM in AGS‐5FU and HGC‐27‐5FU cells (Figure 4C). CircRNAs are demonstrated to have a sponge‐like effect on miRNA by forming a circRNA‐AGO2‐miRNA complex. RIP assay confirmed that AGO2 bound to both circCPM and miR‐21‐3p (Figure 4D,E). Subsequently, FISH assay manifested that circCPM and miR‐21‐3p co‐localized in cytoplasm (Figure 4F).

To investigate the potential mechanisms of circCPM and miR‐21‐3p in autophagy and chemoresistance, co‐transfection in GC cells was applied. CircCPM siRNA and miR‐21‐3p inhibitor were transfected in 5‐FU resistant cells. Apoptosis assays indicated that the apoptosis ratio was greatly improved by circCPM siRNA, which was reduced when co‐transfected with miR‐21‐3p inhibitor (Figure 4H and Figure S1T). The expression of apoptosis‐related protein caspase3 and cleaved caspase3(c‐caspase3) further confirmed the results (Figure 4G and Figure S3A,B). Opposite results were observed in 5‐FU sensitive cells transfected with circCPM plasmid and miR‐21‐3p mimic with or without CQ treatment (Figure 4I,J,N and Figure S1T).

Additionally, western blotting results revealed that circCPM siRNA distinctly inhibited the expression level of LC3; however, the low expression levels of LC3 were rescued in 5‐FU resistant cells when co‐transfected with miR‐21‐3p inhibitor. Another autophagy marker p62 showed the contrary results (Figure 4K and Figure S3C). The opposite effects were observed in 5‐FU sensitive cells transfected with miR‐21‐3p mimic and circCPM overexpressing vectors with or without CQ treatment (Figure 4M and Figure S3D). Moreover, we found that co‐transfection of miR‐21‐3p inhibitor reversed the effect of silencing circCPM on decreasing autophagic level (Figure 4L,O and Figure S3E,G), while co‐transfection of miR‐21‐3p mimic and circCPM overexpressing vectors partially recovered the effect of overexpressing circCPM in 5‐FU sensitive cells with or without CQ treatment (Figure 4P,Q and Figure S3F,H,I). The analysis of TEM had similar results (Figure 4R and Figure S3J).

Collectively, the above results indicated that circCPM regulates GC autophagy and chemoresistance via miR‐21‐3p.

### 3.5 MiR‐21‐3p regulates autophagy and chemoresistance by targeting PRKAA2

**FIGURE 5:**
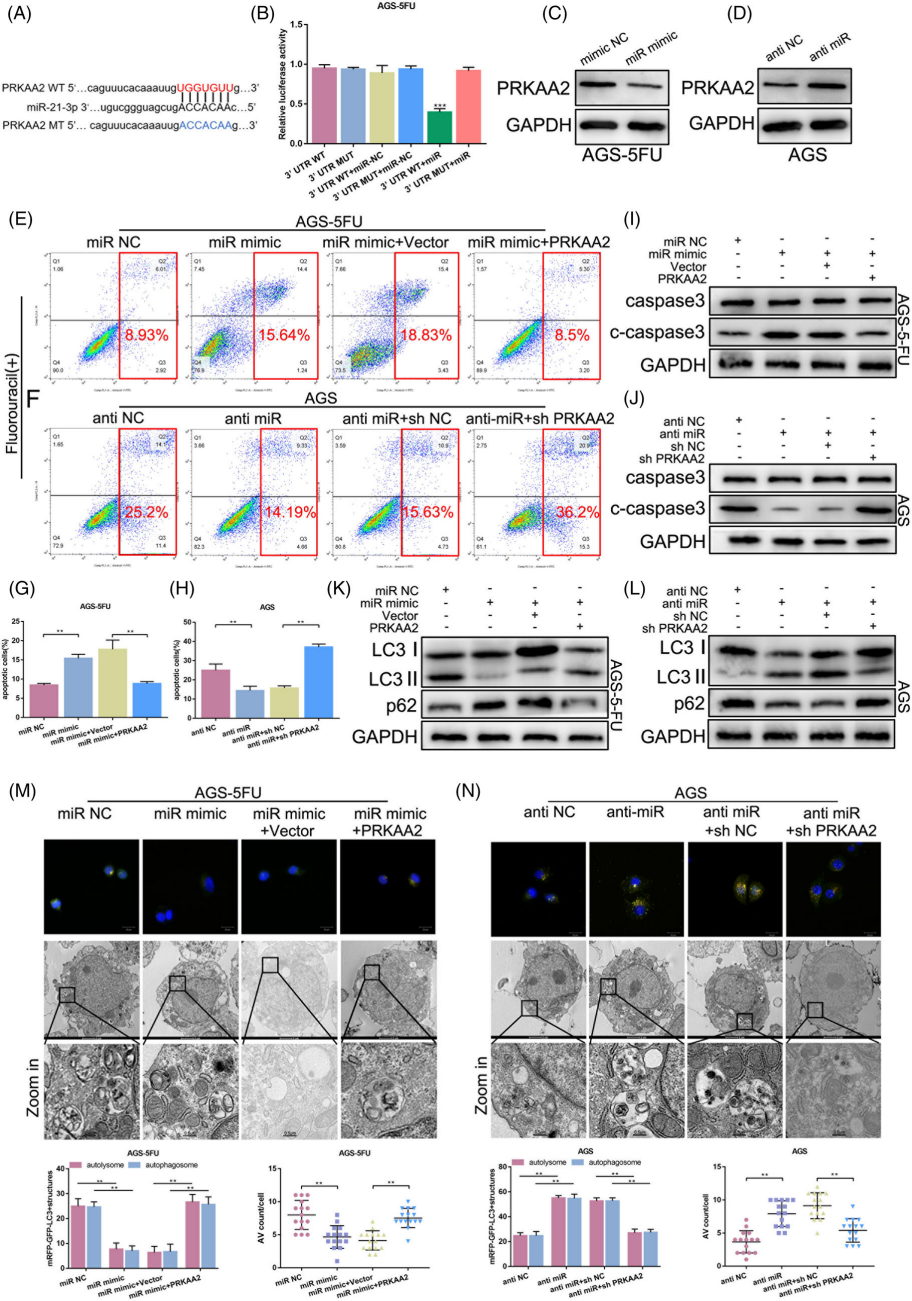
MiR‐21‐3p regulates autophagy and chemoresistance by targeting PRKAA2. (A) Schematic diagram shows the putative‐binding site of miR‐21‐3p with PRKAA2 predicted by TargetScan. (B) Luciferase reporter assay showed that luciferase activity decreased obviously after co‐transfection of miR‐21‐3p with the constructed PRKAA2 3′UTR‐wt plasmid. (C and D) Western blotting analysis of the expression of PRKAA2 in AGS‐5FU and AGS cells transfected with miR mimic and anti‐miR. (E and G) Apoptotic assays of AGS‐5‐FU cells transfected with miR mimic or co‐transfected PRKAA2 overexpression vector upon 5‐FU exposure (25 μM 48 h). (F and H) Apoptotic assays of AGS cells transfected with anti miR or co‐transfected with sh‐PRKAA2 in AGS upon 5‐FU exposure (5 μM 48 h). (I and K) Western blot analysis of caspase3, c‐caspase 3, LC3 and p62 in AGS‐5FU transfected with miR mimic or co‐transfected with PRKAA2 overexpression vector upon 5‐FU exposure (25 μM 48 h). (J and L) Western blot analysis of caspase3, c‐caspase 3, LC3 and p62 in AGS transfected with anti‐miR or co‐transfected with sh‐PRKAA2 in AGS upon 5‐FU exposure (5 μM 48 h). (M) Immunofluorescence analysis (scale bar 20 μm) and transmission electron microscopy (TEM) images (scale bar = 2 μm or 0.5 μm) of AGS‐5FU transfected with miR mimic or co‐transfected PRKAA2 overexpression vector upon 5‐FU exposure (25 μM 48 h). Left lower panel: Quantification data of autolysosome and autophagosome in AGS‐5FU. Right lower panel: Quantification data of autophagic vacuoles (AV) counts in AGS‐5FU. (N) Immunofluorescence analysis (scale bar 20 μm) and TEM images (scale bar = 2 μm or 0.5 μm) of AGS transfected with anti‐miR or co‐transfected with sh‐PRKAA2 upon 5‐FU exposure (5 μM 48 h). Left lower panel: Quantification data of autolysosome and autophagosome in AGS. Right lower panel: Quantification data of AV counts in AGS. The number of AV of 15 cells was counted in each section. (Graph represents mean ± SD; *p < .05, **p < .01 and ***p < .001)

PRKAA2 has been reported to participate in regulating autophagy, which is an important cause of drug resistance. Therefore, we hypothesized that PRKAA2 might be involved in the formation of 5‐FU resistance.^30^ The potential binding sites of PRKKA2 for miR‐21‐3p is 5′UGGUGUU3’ (Figure 5A). Next, a luciferase reporter assay was designed, containing a vector with either wild type sequence or mutant binding site sequence of PRKAA2. Compared to PRKAA2 3′ UTR‐mut, co‐transfection of PRKAA2 3′ UTR‐wt with miR‐21‐3p expression plasmid decreased luciferase activity, which showed the direct binding of miR‐21‐3p on PRKAA2 (Figure 5B). Western blotting further confirmed that PRKAA2 expression was post‐transcriptionally regulated by miR‐21‐3p (Figure 5C,D).

Subsequently, fluorescence activated cell sorting (FACS) and western blotting assay displayed that miR‐21‐3p mimic induced apoptosis in 5‐FU resistant cells. However, co‐transfection of PRKAA2 overexpression vector and miR‐21‐3p mimic abrogated these effects (Figure 5E,G,I and Figure S4A). Opposite results were observed in 5‐FU sensitive cells (Figure 5F,H,J and Figure S4B). Autophagy levels were reduced by overexpressing of miR‐21‐3p while rescued by overexpressing of PRKAA2 in chemoresistant cells (Figure 5K,M and Figure S4C,E). Opposite results were observed in chemosensitive cells (Figure 5L,N and Figure S4D,F).

In summary, miR‐21‐3p regulates GC autophagy and chemoresistance through targeting PRKAA2.

### 3.6 CircRNA regulates chemoresistance of GC through PRKAA2‐mediated autophagy

**FIGURE 6:**
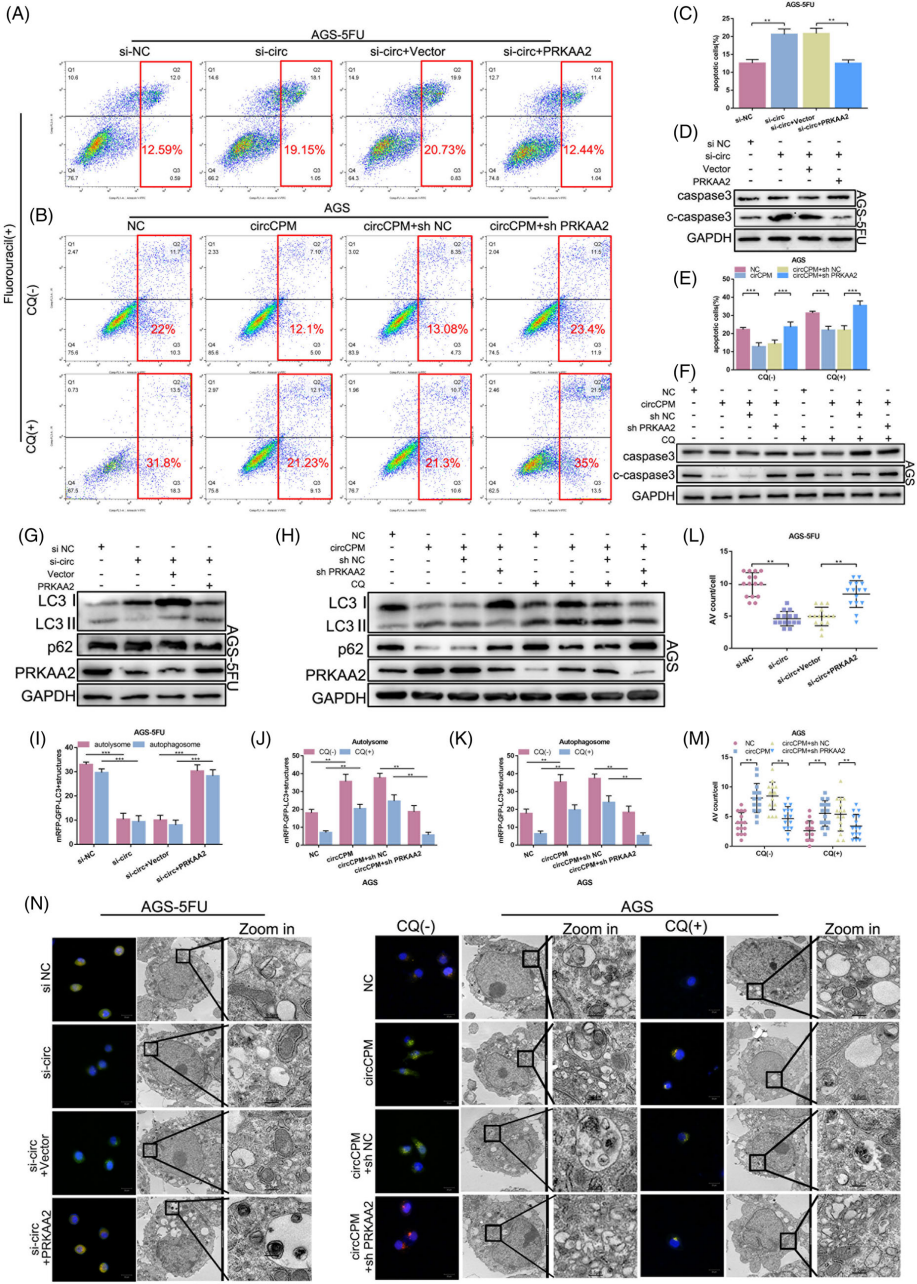
Circular CPM (CircRNA) regulates gastric cancer (GC) chemoresistance through PRKAA2‐mediated autophagy. (A and C) apoptotic assays of AGS‐5FU cells transfected with si‐circ or co‐transfected with PRKAA2 overexpression vector upon 5‐FU (25 μM 48 h) exposure. (B and E) Apoptotic assays of AGS transfected with circCPM overexpression vector or co‐transfected with sh‐PRKAA2 upon 5‐FU (5 μM 48 h) and/or chloroquine (CQ) (20 μM 24 h) exposure. (D and G) Western blot analysis of caspase3, c‐caspase 3, LC3 and p62 in AGS‐5FU transfected with si‐circ or co‐transfected with PRKAA2 overexpression vector upon 5‐FU (25 μM 48 h) exposure. (F and H) Western blot analysis of caspase3, c‐caspase 3, LC3 and p62 in AGS transfected with circCPM overexpression vector or co‐transfected with sh‐PRKAA2 upon 5‐FU (5 μM 48 h) and/or CQ (20 μM 24 h) exposure. (I‐N) Immunofluorescence analysis (scale bar 20 μm) and transmission electron microscopy (TEM) images (scale bar = 2 μm or 0.5 μm) of AGS‐5FU and AGS. (I and L) Quantification data of autolysosome and autophagosome and autophagic vacuoles (AV) counts in AGS‐5FU transfected with si‐circ or co‐transfected with PRKAA2 overexpression vector upon 5‐FU (25 μM 48 h) exposure. (J, K and M) Quantification data of autolysosome and autophagosome and AV counts in AGS transfected with circCPM overexpression vector or co‐transfected with sh‐PRKAA2 upon 5‐FU (5 μM 48 h) and/or CQ (20 μM 24 h) exposure. (Graph represents mean ± SD; *p < .05, **p < .01 and ***p < .001)

We further explored the relationship between circCPM and PRKAA2. Firstly, FACS and Western Blot (WB) revealed that overexpressing PRKAA2 can attenuate the increased apoptotic cells induced by circCPM siRNA (Figure 6A,C,D and Figure S5A). Similarly, the expression of autophagy key proteins, confocal immunofluorescence and TEM assays all indicated that autophagy was recovered by enhanced PRKAA2 expression on the basis of silencing circCPM (Figure 6G,I,L,N and Figure S5C,E,G, Figure S5J).

The similar experiments were performed in 5‐FU sensitive cells by co‐transfecting circCPM plasmid and sh‐PRKAA2. Results showed that sh‐PRKAA2 could rescue the decreased apoptosis level and increased autophagy caused by circCPM overexpression (Figure 6B,D–F,H,J,K,M,N and Figure S5B,D,F, Figure S5H–J).

Taken together, circCPM regulates chemoresistance of GC through PRKAA2‐mediated autophagy.

### 3.7 CircCPM strengthens 5‐FU resistance in vivo

**FIGURE 7:**
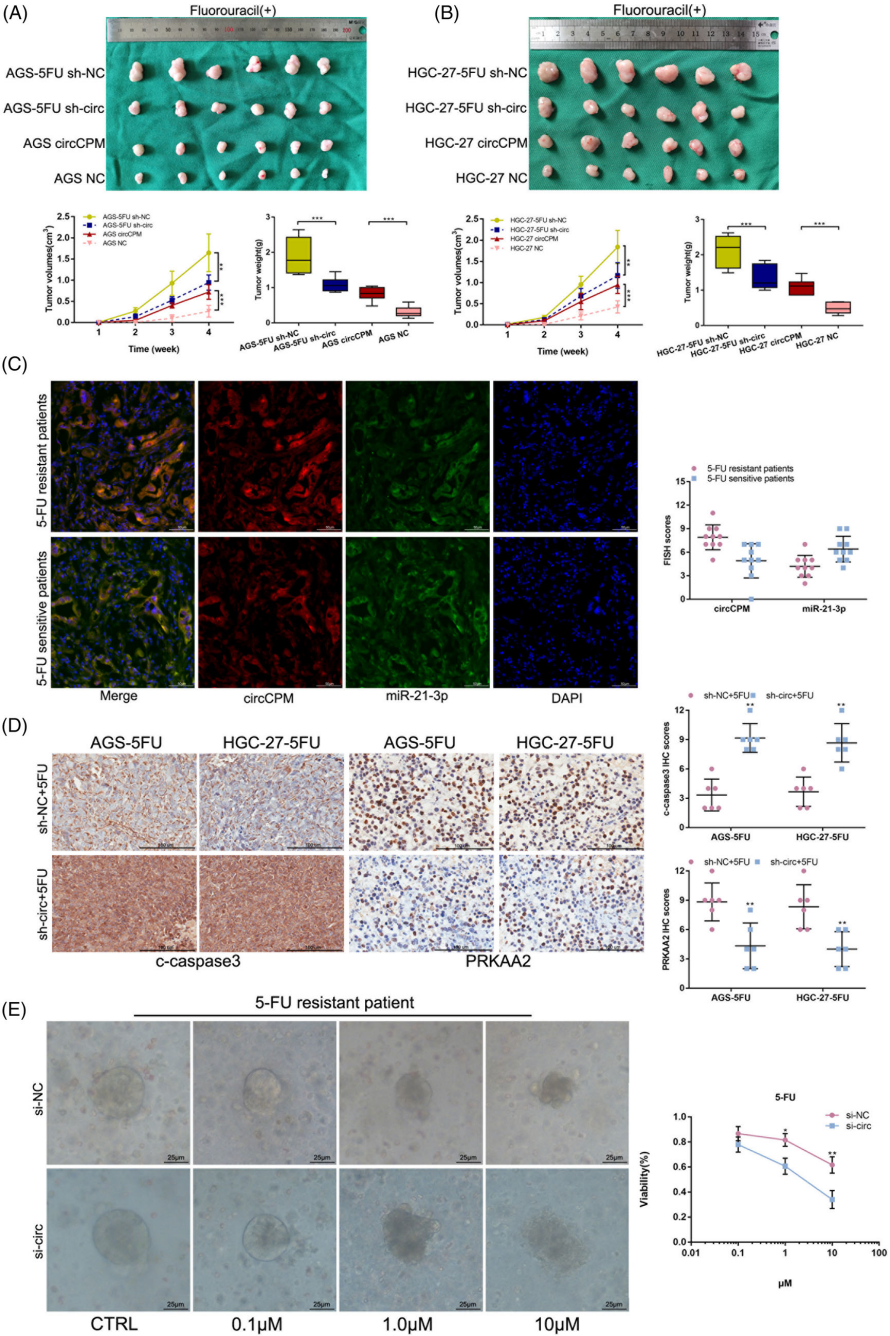
Circular CPM (CircCPM) strengthenes 5‐fluorouracil (5‐FU) resistance in gastric cancer (GC) cells in vivo. (A) Xenograft tumours comprising AGS‐5FU or AGS transfected with sh‐circ or circCPM overexpression vector with 5‐FU treatment (0.5 mg/kg, three times a week) at the end of the experiment. Left lower panel: Tumour volumes were measured weekly in all mice. Right lower panel: Tumours were extracted and weighed. (B) Xenograft tumours comprising HGC‐27‐5FU or HGC‐27 transfected with sh‐circ or circCPM overexpression vector with 5‐FU treatment (0.5 mg/kg, three times a week) at the end of the experiment. Left lower panel: Tumour volumes were measured weekly in all mice. Right lower panel: Tumours were extracted and weighed. (C) Fluorescence in situ hybridization (FISH) showing the co‐localization of circCPM (red) and miR‐21‐3p (green) in 5‐FU‐resistant or 5‐FU‐sensitive GC tissues from patients. FISH scores of circCPM and miR‐21‐3p were further calculated in 10 5‐FU‐resistant and 10 5‐FU‐sensitive patient tissues. Nuclei were stained with 4,6‐diamino‐2‐phenyl indole (DAPI). Scale bar = 10 μm. (D) Immunohistochemical staining against c‐caspase3 and PRKAA2 were used to determine the effects of circCPM on c‐caspase3 and PRKAA2 in xenograft tumours. Scale bar = 200 μm. (E) Representative picture and cell viability assay of GC organoid transfected with si‐circ after treatment with different concentrations of 5‐FU (48 h). Scale bar = 25 μm. (Graph represents mean ± SD; *p < .05, **p < .01 and ***p < .001)

To further evaluate the clinical value of circCPM, cells with silencing or overexpressing circCPM were subcutaneously injected into BALB/c nude mice in conjunction with chemotherapy drugs and allowed to proliferate for 4 weeks. Tumours were weighed and measured separately. Results indicated that silencing circCPM in 5‐FU resistant cells hugely decreased xenograft tumour weight and volume and enhanced the effects of 5‐FU treatment in GC, while overexpressing circCPM showed the opposite results (Figure 7A,B). FISH showed the circCPM and miR‐21‐3p were co‐localized in tissues from 5‐FU resistant or 5‐FU sensitive GC patients. FISH assay showed circCPM expression was higher in 5‐FU resistant GC tissues (Figure 7C). Elevated protein levels of c‐caspase3 were observed by IHC in tumour transfected with sh‐circCPM combined with 5‐FU chemotherapy than the control group, while PRKAA2 displayed the opposite results (Figure 7D). We also established an organoid model to observe chemotherapy sensibility of 5‐FU. Cell viability assays documented that organoids transfected with the circCPM siRNA had lower cell activity. Morphologically, responding organoids became dark and disaggregated (Figure 7E).

Overall, circCPM promotes GC 5‐FU chemoresistance in vivo.

## 4 DISCUSSION

The distribution of GC in the world has prominent regional characteristics, and about 50% of GC patients are distributed in East Asia.^4^ In China, more than 80% of patients have been the advanced stage at the time of diagnosis, contributing to a very low 5‐year survival rate of only 25%.^31^ Chemotherapy is the recommended treatment for advanced GC patients with or without radical resection.^32^, ^33^ Currently, 5‐FU is one of the first‐line chemotherapy agents for advanced GC, which is widely applied in the clinic. However, 5‐FU resistance leads to poor chemotherapy efficacy and patients’ prognosis.^14^, ^15^, ^16^ Several factors contribute to 5‐FU chemoresistance, including deficient drug transport mechanisms, alterations of target enzymes, activation of DNA repair pathways, resistance to apoptosis, changes in the tumour microenvironment, and other serious problems have been reported.^13^

Autophagy acts as an essential homeostatic and catabolic process, playing a vital role in several cellular functions, including tumour formation and resistance to cancer therapy.^19^, ^20^ Emerging evidence confirms that excessive activation of autophagy can safeguard tumours against apoptosis induced by various factors, including anticancer drugs.^34^, ^35^ This may provide new thinking that suppression of autophagy may be able to reverse tumour chemoresistance. To clarify the relationship between autophagy and chemoresistance, we have already carried out several studies and identified several non‐coding RNAs. In this study, to further clarify the possible mechanism of autophagy on 5‐FU resistance in GC, microarray, miRNA sequencing and bioinformatics analysis were performed. Autophagy‐related gene PRKAA2 was found to be up‐regulated in 5‐FU resistant cells and tissues and associated with lower overall survival of patients who received 5‐FU‐based chemotherapy. PRKAA2 knockdown inhibited autophagy and promoted apoptosis and chemosensitivity of GC cells to 5‐FU in virto and in vivo. Further analysis and experiments probed an autophagy‐related circCPM‐miR‐21‐3p‐PRKAA2 axis. As far as we know, this is the first study in clarifying the role of PRKAA2 in GC 5‐FU resistance.

In mammals, AMPK is a serine/threonine kinase consisting of α, β and γ subunits.^36^ AMPKα contains two isoforms α1 and α2. Both isoforms are closely related to autophagy. AMPKα1 activates autophagy by directly phosphorylating ULK1, BECN1 and Vps34, key autophagy‐related proteins.^37^, ^38^ AMPKα2 (also termed as PRKAA2) mediates autophagy by transcriptionally regulating several autophagy‐related genes, including SKP2‐CARM1 signaling cascade.^30^ Besides, AMPKα2 can regulate autophagy by directly elevating ULK1 activity and indirectly reducing mTOR activity to induce autophagy.^39^ Previous studies have proved that PRKAA2 is regulated by several miRNAs, including miR‐124‐3p and miR‐4999‐5p.^40^, ^41^ In this study, we first identified that PRKAA2 is downstream of miR‐21‐3p. It is proved that circCPM could significantly promote PRKAA2 expression; thus, circCPM‐up‐regulated GC may have an increased ability for 5‐FU resistance through increased autophagy. It is noteworthy that although we have proved that circCPM functions as a regulator of PRKAA2 via miR‐21‐3p, there may have other regulatory mechanisms, such as post‐translational regulation. For example, L.‐Y. Li et al. reported that PRKAA2 could be phosphorylated by RSK2 at Thr172 residue.^42^ In addition, PRKAA2 was reported to take part in regulating metabolic phenotype, including glucose and fatty acid metabolism.^43^ Moreover, metabolic reprogramming has been reported to participate in chemoresistance.^44^ However, whether metabolic phenotype switch influences 5‐FU resistance in GC remains unknown.

Abundant evidence shows that circRNAs play important roles in a variety of cellular processes, including chemoresistance.^25^, ^26^ However, chemoresistance‐associated circRNAs in GC 5‐FU resistance have rarely been reported. Here, we found the higher expression of circCPM in chemoresistant tissues and cells through ceRNA arrays. Follow‐up data analysis indicated that circCPM was correlated with the overall survival of patients receiving 5‐FU chemotherapy. Various experiments proved circCPM could promote 5‐FU resistance via autophagy in GC. Silencing circCPM could promote apoptosis and improve chemotherapy sensitivity in virto. The data of xenograft and organoid model in vivo showed that silencing circCPM in 5‐FU resistant cells hugely limited xenograft tumour growth and reduced tumour cell activity. Besides, we found that circCPM and CPM mRNA are both up‐regulated in GC cells or tissues. According to previous studies, linear CPM may serve as a potentially predictive serum biomarker, possibly suggesting that underlying connections may exist between the linear CPM and 5‐FU chemoresistance. The upstream regulatory mechanism of circCPM and CPM mRNA needs further exploration in our future research.

The most‐known function patterns for circRNAs are working as miRNA sponges, regulating transcription of genes in the nucleus and encoding proteins.^23^, ^24^ Considering the exonic sequence, distribution and abundance of circCPM, we selected a ‘sponging’ model for circCPM and miR‐21‐3p to interact in GC. This mechanism has been verified in several studies we carried out before. Wang et al. reported circOSBPL10 ‘sponges’ miR‐136‐5p to promote GC cell proliferation and migration.^45^ Through bioinformatics prediction, luciferase reporter assay and RNA pull down assays, we proved that circCPM showed a sponge‐like effect on miR‐21‐3p. Many studies have reported that miR‐21‐3p participates in a variety of diseases including cancer, atherosclerosis and so on. For instance, Gao et al. reported that hsa‐miR‐21‐3p affects cell stemness in esophageal squamous cell carcinoma.^46^ Zhu J et al. reported that exosomes containing miR‐21‐3p accelerates atherosclerosis through regulating phosphatase and tension homologue (PTEN)‐mediated vascular smooth muscle cells (VSMC) migration and proliferation.^47^ In this study, we confirmed that circCPM regulated 5‐FU resistance in GC by targeting miR‐21‐3p, which in turn enhanced autophagy by increasing PRKAA2 translation.

## 5 CONCLUSION

We performed functional experiments and adopted several models to prove that circCPM is up‐regulated in 5‐FU resistant GC cell lines and tissues and induces GC 5‐FU chemoresistance by working as a sponge of miR‐21‐3p, thereby up‐regulating PRKAA2 expression. Besides, circCPM functions as a valuable prognostic factor in GC 5‐FU resistance. All the results indicate that circCPM could be a biomarker for 5‐FU resistance and a target to overcome drug resistance in GC.

## CONFLICT OF INTEREST

The authors declare no competing interests.

## Supporting information



Supporting Information

**Figure S1** (A) CCK8 assay of the effect of silencing PRKAA2 on the drug sensitivity of HGC‐27‐5FU cells. (B‐F) CCK8 assay of the effect of silencing five‐candidate circRNAs on the drug sensitivity of HGC‐27‐5FU cells. (G‐J) Quantitative Real‐time PCR (qRT‐PCR) analysis of efficiency of knockdown and overexpression of circCPM. (K) Apoptotic assays of AGS‐5FU cells transfected with si‐circ or co‐transfected anti‐miR upon 5‐FU exposure (25 μM 48 h) and AGS cells transfected with circCPM overexpression vector or co‐transfected with miR mimic upon 5‐FU (5 μM 48 h) and/or CQ (20 μM 24 h) exposure. (Graph represents mean ± SD; *p < .05, **p < .01 and ***p < .001.)

**Figure S2** (A) CCK8 assay of the effect of overexpressing circCPM on the drug sensitivity of GC cells. (B) Colony formation assays of HGC‐27‐5FU and HGC‐27 cells were performed to assess the proliferative ability. Right upper panel: Quantification data for HGC‐27‐5FU transfected with si‐circ with or without 5‐FU exposure (30 μM 48 h). Right lower panel: Quantification data for HGC‐27 transfected with circCPM overexpression plasmids with or without 5‐FU (6 μM 48 h) and/or CQ (20 μM 24 h) exposure. (C) Apoptotic assays of GC cells to assess circCPM modulation on the drug sensitivity. Right upper panel: Quantification data for HGC‐27‐5FU transfected with si‐circ with or without 5‐FU exposure (30 μM 48 h). Right lower panel: Quantification data for HGC‐27 transfected with circCPM overexpression plasmids with or without 5‐FU (6 μM 48 h) and/or CQ (20 μM 24 h) exposure. (D, G and H) Immunofluorescence analysis using Green fluorescent protein (GFP)‐Monomeric Red Fluorescent Protein (mRFP)‐LC3 staining. Scale bar 10 μm. (G and H) The numbers of LC3 puncta were quantified in HGC‐27‐5FU transfected with si‐circ upon 5‐FU exposure (30 μM 48 h) and HGC‐27 transfected with circCPM overexpression plasmids upon 5‐FU (6 μM 48 h) and/or CQ (20 μM 24 h) exposure. (E) Western blot analysis of LC3 and p62 protein levels in cells transfected with si‐circ or circCPM overexpression in HGC‐27‐5FU and HGC‐27. (F, I and J) TEM images of ultrastructure microstructure in representative HGC‐27‐5FU transfected with si‐circ upon 5‐FU exposure (30 μM 48 h) and HGC‐27 cells transfected with circCPM overexpression plasmids upon 5‐FU (6 μM 48 h) and/or CQ (20 μM 24 h) exposure. (I and J) The number of autophagic vacuoles (AV) of 15 cells was counted in each section. Scale bar = 2 μm or 0.5 μm. (Graph represents mean ± SD; *p < .05, **p < .01 and ***p < .001)

**Figure S3** (A and C) Western blot analysis of caspase3, c‐caspase 3, LC3 and p62 inHGC‐27‐5FU transfected with si‐circ or co‐transfected with anti‐miR upon 5‐FU exposure (30 μM 48 h). (B and D) Western blot analysis of caspase3, c‐caspase 3, LC3 and p62 in HGC‐27 transfected with circCPM overexpression vector or co‐transfected with miR mimic upon 5‐FU exposure (6 μM 48 h). (E and G) Immunofluorescence analysis of HGC‐27‐5FU transfected with si‐circ or co‐transfected anti‐miR upon 5‐FU exposure (30 μM 48 h). (G) quantification data of autolysosome and autophagosome in HGC‐27‐5FU. Scale bar 10 μm. (F, H and I) Immunofluorescence analysis of HGC‐27 transfected with si‐circ or co‐transfected anti‐miR upon 5‐FU (6 μM 48 h) and/or CQ (20 μM 24 h) exposure. (H and I) Quantification data of autolysosome and autophagosome in HGC‐27. Scale bar 10 μm. (J) TEM images of HCG‐27‐5‐FU and HGC‐27 with specific treatments. Scale bar = 2 μm or 0.5 μm. Left lower panel: Quantification data of AV counts in HGC‐27‐5FU transfected with si‐circ or co‐transfected anti‐miR upon 5‐FU exposure (30 μM 48 h). Right lower panel: Quantification data of AV counts in HGC‐27 transfected with si‐circ or co‐transfected anti‐miR upon 5‐FU (6 μM 48 h) and/or CQ (20 μM 24 h) exposure. The number of AV of 15 cells was counted in each section. (Graph represents mean ± SD; *p < .05, **p < .01 and ***p < .001)

**Figure S4** (A and C) Western blot analysis of caspase3, c‐caspase 3, LC3 and p62 in HGC‐27‐5FU transfected with miR mimic or co‐transfected with PRKAA2 overexpression vector upon 5‐FU exposure (30 μM 48 h). (B and D) Western blot analysis of caspase3, c‐caspase 3, LC3 and p62 in HGC‐27 transfected with anti‐miR or co‐transfected with sh‐PRKAA2 in HGC‐27 upon 5‐FU exposure (6 μM 48 h). (E) Immunofluorescence analysis and TEM images of HGC‐27‐5FU transfected with miR mimic or co‐transfected PRKAA2 overexpression vector upon 5‐FU exposure (30 μM 48 h). Scale bar 10 μm. Left lower panel: Quantification data of autolysosome and autophagosome in HGC‐27‐5FU. Right lower panel: Quantification data of AV counts in HGC‐27‐5FU. (F) Immunofluorescence analysis (scale bar 10 μm) and TEM images (scale bar = 2 μm or 0.5 μm) of HGC‐27 transfected with anti‐miR or co‐transfected with sh‐PRKA A2 upon 5‐FU exposure (6 μM 48 h). Left lower panel: Quantification data of autolysosome and autophagosome in HGC‐27. Right lower panel: Quantification data of AV counts in HGC‐27. The number of AV of 15 cells was counted in each section. (Graph represents mean ± SD; *p < .05, **p < .01 and ***p < .001)

**Figure S5** (A and C) Western blot analysis of caspase3, c‐caspase 3, LC3 and p62 in HGC‐27‐5FU transfected with si‐circ or co‐transfected with PRKAA2 overexpression vector upon 5‐FU (30 μM 48 h). (B and D) Western blot analysis of caspase3, c‐caspase 3, LC3 and p62 in HGC‐27 transfected with circCPM overexpression vector or co‐transfected with sh‐PRKAA2 upon 5‐FU (6 μM 48 h) and/or CQ (20 μM 24 h) exposure. (E and G) Immunofluorescence analysis of HGC‐27‐5FU transfected with si‐circ or co‐transfected with PRKAA2 overexpression vector upon 5‐FU (30 μM 48 h). Scale bar 10 μm. (G) quantification data of autolysosome and autophagosome. (F, H and I) Immunofluorescence analysis of HGC‐27 transfected with circCPM overexpression vector or co‐transfected with sh‐PRKAA2 upon 5‐FU (6 μM 48 h) and/or CQ (20 μM 24 h) exposure. Scale bar 10 μm. (H and I) Quantification data of autolysosome and autophagosome. (J) TEM images of HCG‐27‐5‐FU and HGC‐27 with specific treatments. Scale bar = 2 μm or 0.5 μm. Left lower panel: Quantification data of AV counts in HGC‐27‐5FU transfected with si‐circ or co‐transfected with PRKAA2 overexpression vector upon 5‐FU (30 μM 48 h). Right lower panel: Quantification data of AV counts in HGC‐27 transfected with circCPM overexpression vector or co‐transfected with sh‐PRKAA2 upon 5‐FU (6 μM 48 h) and/or CQ (20 μM 24 h) exposure. (Graph represents mean ± SD; *p < .05, **p < .01 and ***p < .001)
