# Supplementary material for: Circular CPM promotes chemoresistance of gastric cancer via activating PRKAA2‐mediated autophagy
Source: Clin Transl Med. 2022 Jan 24;12(1):e708. doi: 10.1002/ctm2.708 (PMC8787023; doi:10.1002/ctm2.708)
Supplement: Supplementary file 1 — Supporting Information [file CTM2-12-e708-s004.docx]

**Supplementary figure legends**

**Figure S1**

1. CCK8 assay of the effect of silencing PRKAA2 on the drug sensitivity of HGC-5FU cells.

(B-E) Kaplan–Meier analysis of the correlation between candidate circRNAs expression and overall survival.

(F-N) CCK8 assay of the effect of silencing five candidate circRNAs on the drug sensitivity of 5FU-resistant cells.

(O) qRT-PCR analysis in HGC-27-5FU cells confirming that circular and linear CPM are mainly located in the cytoplasm.

(P-S) qRT-PCR analysis of efficiency of knockdown and overexpression of circCPM.

(T) apoptotic assays of AGS-5FU cells transfected with si-circ or co-transfected anti miR upon 5-FU exposure (25μM 48h) and AGS cells transfected with circCPM overexpression vector or co-transfected with miR mimic upon 5-FU (5μM 48h) and/or CQ (20μM 24h) exposure.

(Graph represents mean ± SD; *p < 0.05, **p < 0.01, and ***p < 0.001.)

**Figure S2**

(A) CCK8 assay of the effect of overexpressing circCPM on the drug sensitivity of GC cells.

(B) colony formation assays of HGC-27-5FU and HGC-27 cells were performed to assess the proliferative ability.

Right upper panel: quantification data for HGC-27-5FU transfected with si-circ with or without 5-FU exposure (30μM 48h).

Right lower panel: quantification data for HGC-27 transfected with circCPM overexpression plasmids with or without 5-FU (6μM 48h) and/or CQ (20μM 24h) exposure.

(C) apoptotic assays of GC cells to assess circCPM modulation on the drug sensitivity. Right upper panel: quantification data for HGC-27-5FU transfected with si-circ with or without 5-FU exposure (30μM 48h).

Right lower panel: quantification data for HGC-27 transfected with circCPM overexpression plasmids with or without 5-FU (6μM 48h) and/or CQ (20μM 24h) exposure.

(D, G, H) Immunofluorescence analysis using GFP-mRFP-LC3 staining. Scale bar 10μm. G, H: The numbers of LC3 puncta were quantified in HGC-27-5FU transfected with si-circ upon 5-FU exposure (30μM 48h) and HGC-27 transfected with circCPM overexpression plasmids upon 5-FU (6μM 48h) and/or CQ (20μM 24h) exposure.

(E) Western blot analysis of LC3 and p62 protein levels in cells transfected with si-circ or circCPM overexpression in HGC-27-5FU and HGC-27.

(F, I, J) TEM images of ultrastructure microstructure in representative HGC-27-5FU transfected with si-circ upon 5-FU exposure (30μM 48h) and HGC-27 cells transfected with circCPM overexpression plasmids upon 5-FU (6μM 48h) and/or CQ (20μM 24h) exposure. I, J: The number of autophagic vacuoles (AV) of 15 cells was counted in each section. Scale bar= 2μm or 0.5μm.

(Graph represents mean ± SD; *p < 0.05, **p < 0.01, and ***p < 0.001.)

**Figure S3**

(A, C) Western blot analysis of caspase3, c-caspase 3, LC3 and p62 in HGC-27-5FU transfected with si-circ or co-transfected with anti miR upon 5-FU exposure (30μM 48h).

(B, D) Western blot analysis of caspase3, c-caspase 3, LC3 and p62 in HGC-27 transfected with circCPM overexpression vector or co-transfected with miR mimic upon 5-FU exposure (6μM 48h).

(E, G) Immunofluorescence analysis of HGC-27-5FU transfected with si-circ or co-transfected anti miR upon 5-FU exposure (30μM 48h). G: quantification data of autolysosome and autophagosome in HGC-27-5FU. Scale bar 10μm.

(F, H, I) Immunofluorescence analysis of HGC-27 transfected with si-circ or co-transfected anti miR upon 5-FU (6μM 48h) and/or CQ (20μM 24h) exposure. H, I: quantification data of autolysosome and autophagosome in HGC-27. Scale bar 10μm.

(J) TEM images of HCG-27-5FU and HGC-27 with specific treatments. Scale bar= 2μm or 0.5μm.

Left lower panel: quantification data of AV counts in HGC-27-5FU transfected with si-circ or co-transfected anti miR upon 5-FU exposure (30μM 48h).

Right lower panel: quantification data of AV counts in HGC-27 transfected with si-circ or co-transfected anti miR upon 5-FU (6μM 48h) and/or CQ (20μM 24h) exposure. The number of AV of 15 cells was counted in each section.

(Graph represents mean ± SD; *p < 0.05, **p < 0.01, and ***p < 0.001.)

**Figure S4**

(A, C) Western blot analysis of caspase3, c-caspase 3, LC3 and p62 in HGC-27-5FU transfected with miR mimic or co-transfected with PRKAA2 overexpression vector upon 5-FU exposure (30μM 48h).

(B, D) Western blot analysis of caspase3, c-caspase 3, LC3 and p62 in HGC-27 transfected with anti miR or co-transfected with sh PRKAA2 in HGC-27 upon 5-FU exposure (6μM 48h).

(E) Immunofluorescence analysis and TEM images of HGC-27-5FU transfected with miR mimic or co-transfected PRKAA2 overexpression vector upon 5-FU exposure (30μM 48h). Scale bar 10 μm.

Left lower panel: quantification data of autolysosome and autophagosome in HGC-27-5FU

Right lower panel: quantification data of AV counts in HGC-27-5FU.

(F) Immunofluorescence analysis (Scale bar 10μm.) and TEM images (Scale bar= 2μm or 0.5μm) of HGC-27 transfected with anti miR or co-transfected with sh PRKA­­­A2 upon 5-FU exposure (6μM 48h).

Left lower panel: quantification data of autolysosome and autophagosome in HGC-27.

Right lower panel: quantification data of AV counts in HGC-27. The number of AV of 15 cells was counted in each section.

(Graph represents mean ± SD; *p < 0.05, **p < 0.01, and ***p < 0.001.)

**Figure S5**

(A, C) Western blot analysis of caspase3, c-caspase 3, LC3 and p62 in HGC-27-5FU transfected with si-circ or co-transfected with PRKAA2 overexpression vector upon 5-FU (30μM 48h).

(B, D) Western blot analysis of caspase3, c-caspase 3, LC3 and p62 in HGC-27 transfected with circCPM overexpression vector or co-transfected with sh PRKAA2 upon 5-FU (6μM 48h) and/or CQ (20μM 24h) exposure.

(E, G) Immunofluorescence analysis of HGC-27-5FU transfected with si-circ or co-transfected with PRKAA2 overexpression vector upon 5-FU (30μM 48h). Scale bar 10μm.

G: quantification data of autolysosome and autophagosome.

(F, H, I) Immunofluorescence analysis of HGC-27 transfected with circCPM overexpression vector or co-transfected with sh PRKAA2 upon 5-FU (6μM 48h) and/or CQ (20μM 24h) exposure. Scale bar 10μm.

H, I: quantification data of autolysosome and autophagosome

(J) TEM images of HCG-27-5FU and HGC-27 with specific treatments. Scale bar= 2μm or 0.5μm.

Left lower panel: quantification data of AV counts in HGC-27-5FU transfected with si-circ or co-transfected with PRKAA2 overexpression vector upon 5-FU (30μM 48h).

Right lower panel: quantification data of AV counts in HGC-27 transfected with circCPM overexpression vector or co-transfected with sh PRKAA2 upon 5-FU (6μM 48h) and/or CQ (20μM 24h) exposure.

(Graph represents mean ± SD; *p < 0.05, **p < 0.01, and ***p < 0.001.)

**Supplementary Table 1**

| GAPDH | Forward: TGTACCATCAATAAAGTACCCTGTG |
| --- | --- |
|  | Reverse: AAATCCGTTGACTCCGACCT |
| U6 | Forward: CTCGCTTCGGCAGCACA |
|  | Reverse: AACGCTTCACGAATTTGCGT |
| 18s | Forward: TTAATTCCGATAACGAACGAGA |
|  | Reverse: CGCTGAGCCAGTCAGTGTAG |
| CPM | Forward: TACCACCGCCAGGAAGGG |
|  | Reverse: CCTTTGGAAACCGCCCC |
| hsa-circ-0027497 | Forward: CAAACCTCCATGGTGGTGCCC |
|  | Reverse: CGGGGAAATTTCGATTCAAGTC |
| hsa-circ-0076305 | Forward: CCACTCTGGATTCAGCCTTC |
|  | Reverse: GGTGTCAAAAAGGACCAGGA |
| hsa-circ-0007734 | Forward: TTTCCGGAGATGGAAGTCAC |
|  | Reverse: AACACGGTTCAACACCAGTTT |
| hsa-circ-0065881 | Forward: CAGCATCTTCTCCATCGTCA |
|  | Reverse: CCAGCAAAGTGCTTCTGGAT |
| hsa-circ-0034115 | Forward: CCTGGACATACATGGTGTGG |
|  | Reverse: TGAAGAGGCAGGACATTTGA |
| MiR-21-3p | 5′-CAACAGCAGTCGATGGGCTGT-3′ |
| PRKAA2 | Forward: GTGAAGATCGGACACTACGTG |
|  | Reverse: CTGCCACTTTATGGCCTGTTA |
| Si-circ0027497-1 | sense（5'-3'）CCACUCCAAGACUGUUGGG |
|  | antisense（5'-3'）CCCAACAGUCUUGGAGUGG |
| Si-circ0027497-2 | sense（5'-3'）GUAUCCACUCCAAGACUGU |
|  | antisense（5'-3'）ACAGUCUUGGAGUGGAUAC |
| Sh PRKAA2 | 5'CACCGCAGCTTCTGGTCAAACAAATCTCGAGATTTGTTTGACCAGAAGCTGC 3' |
|  | 5'AAAAGCAGCTTCTGGTCAAACAAATCTCGAGATTTGTTTGACCAGAAGCTGC 3' |

**Supplementary Table 2**

| GAPDH | ab8245 | Abcam |
| --- | --- | --- |
| LC3 | 14600-1-AP | Proteintech |
| p62 | 18420-1-AP | Proteintech |
| caspase3 | ab32351 | Abcam |
| c-caspase3 | ab2302 | Abcam |
| c-caspase3 | #9664 | CST |
| PRKAA2 | ab3760 | Abcam |
