# Supplementary figures and images for: Circular CPM promotes chemoresistance of gastric cancer via activating PRKAA2‐mediated autophagy
Source: Clin Transl Med. 2022 Jan 24;12(1):e708. doi: 10.1002/ctm2.708 (PMC8787023; doi:10.1002/ctm2.708)

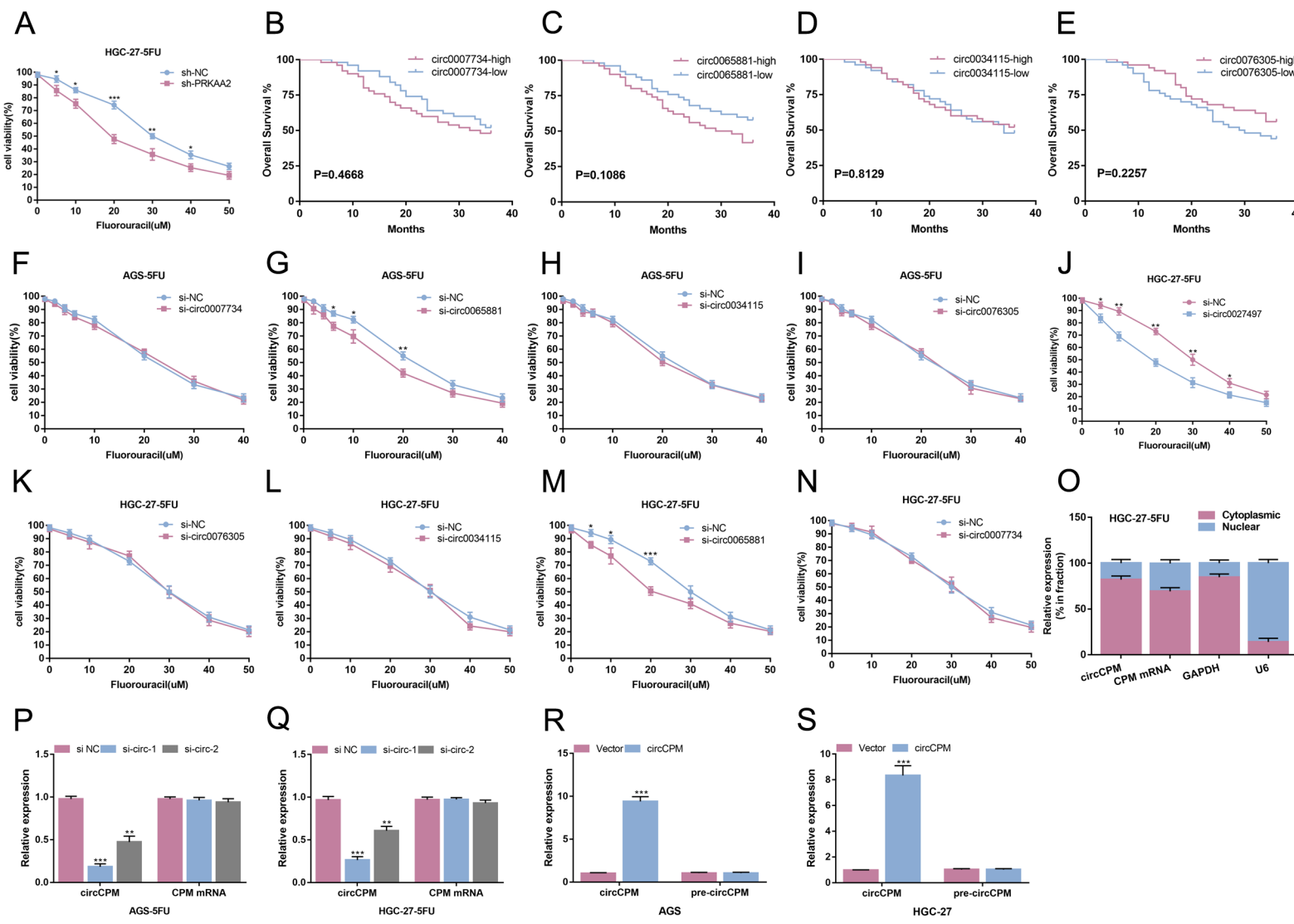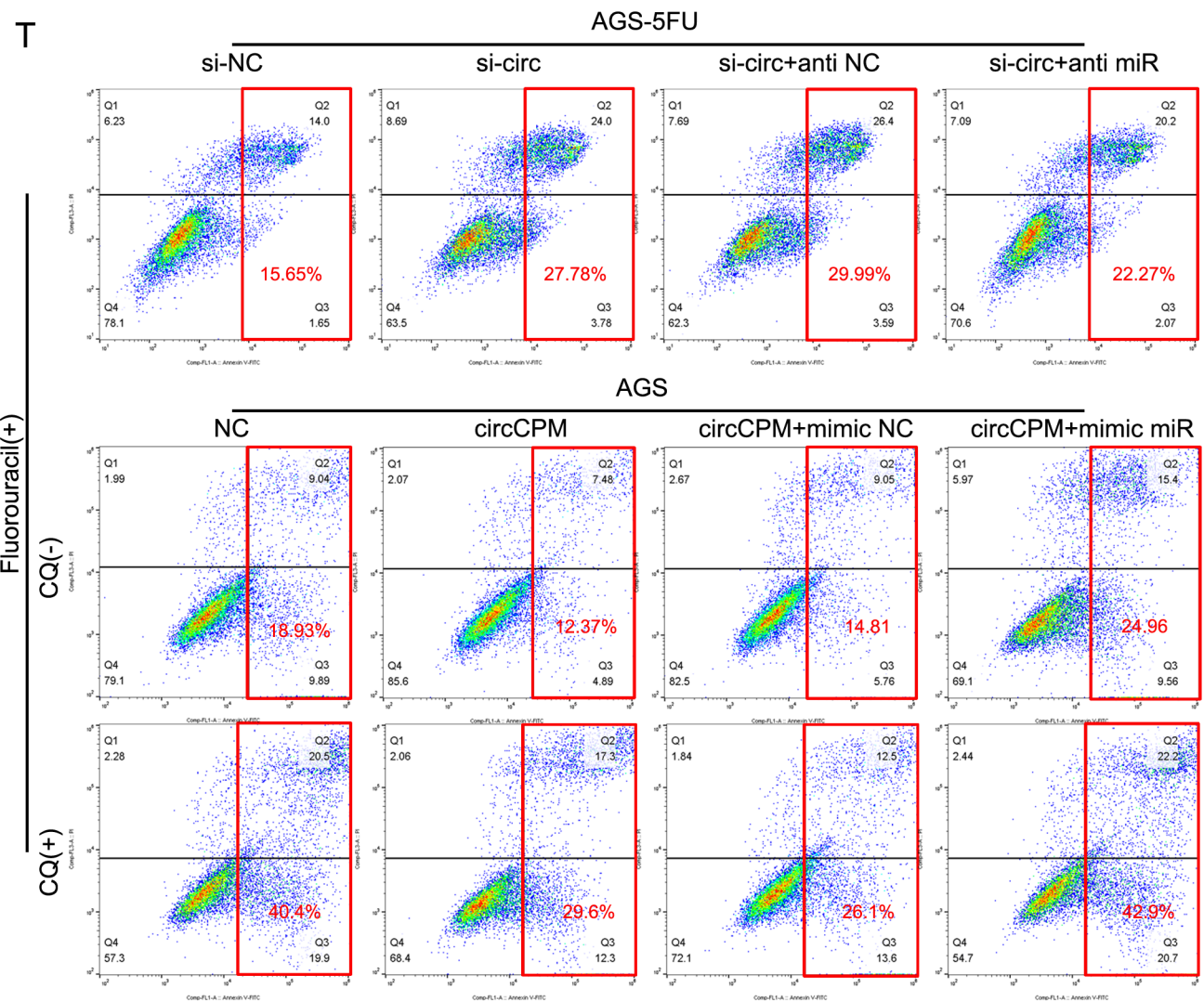

Supplement: Supplementary file 2 — Figure S1 (A) CCK8 assay of the effect of silencing PRKAA2 on the drug sensitivity of HGC‐27‐5FU cells. (B‐F) CCK8 assay of the effect of silencing five‐candidate circRNAs on the drug sensitivity of HGC‐27‐5FU cells. (G‐J) Quantitative Real‐time PCR (qRT‐PCR) analysis of efficiency of knockdown and overexpression of circCPM. (K) Apoptotic assays of AGS‐5FU cells transfected with si‐circ or co‐transfected anti‐miR upon 5‐FU exposure (25 μM 48 h) and AGS cells transfected with circCPM overexpression vector or co‐transfected with miR mimic upon 5‐FU (5 μM 48 h) and/or CQ (20 μM 24 h) exposure. (Graph represents mean ± SD; *p < .05, **p < .01 and ***p < .001.) [file CTM2-12-e708-s002.pdf]

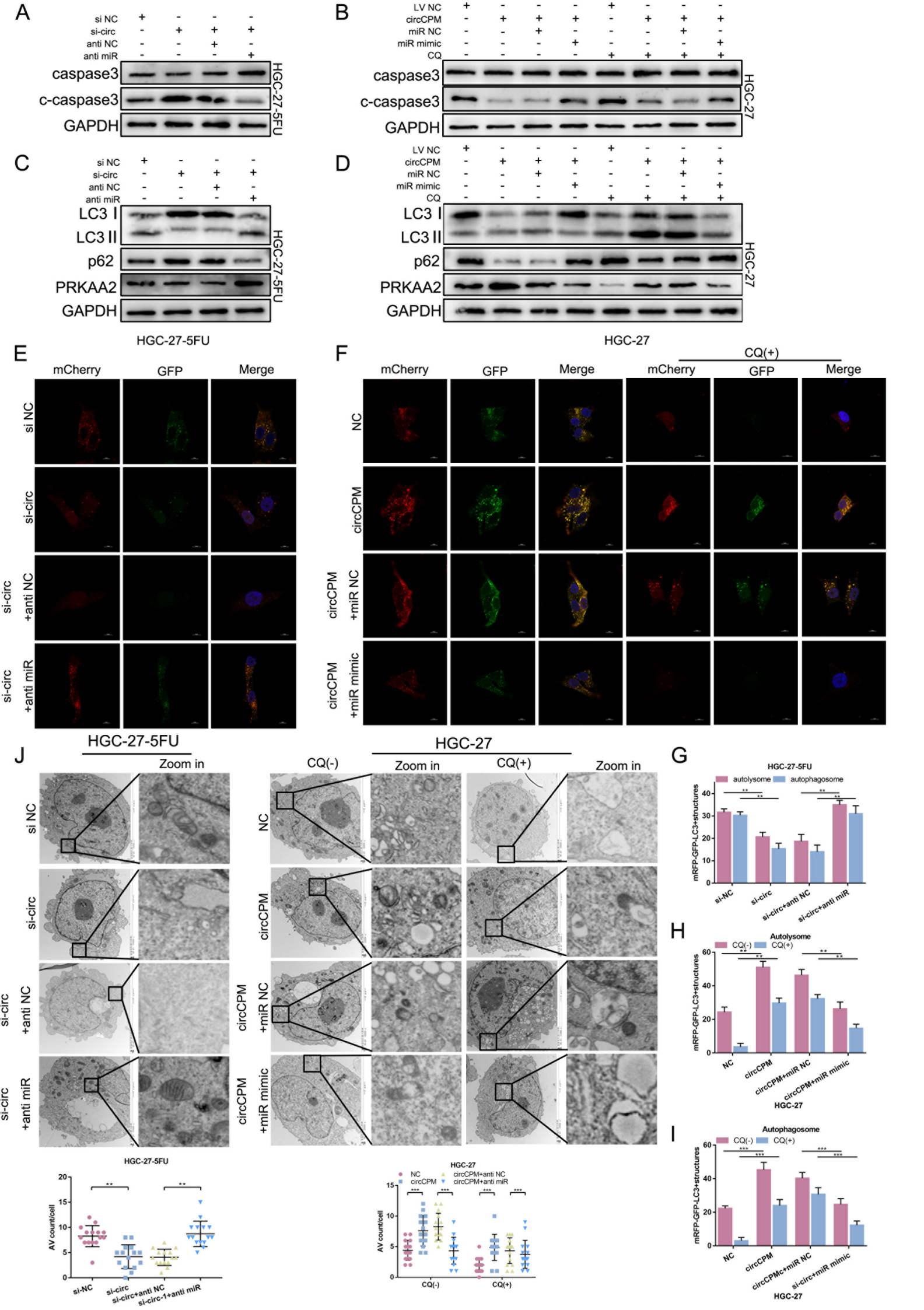

Supplement: Supplementary file 4 — Figure S3 (A and C) Western blot analysis of caspase3, c‐caspase 3, LC3 and p62 inHGC‐27‐5FU transfected with si‐circ or co‐transfected with anti‐miR upon 5‐FU exposure (30 μM 48 h). (B and D) Western blot analysis of caspase3, c‐caspase 3, LC3 and p62 in HGC‐27 transfected with circCPM overexpression vector or co‐transfected with miR mimic upon 5‐FU exposure (6 μM 48 h). (E and G) Immunofluorescence analysis of HGC‐27‐5FU transfected with si‐circ or co‐transfected anti‐miR upon 5‐FU exposure (30 μM 48 h). (G) quantification data of autolysosome and autophagosome in HGC‐27‐5FU. Scale bar 10 μm. (F, H and I) Immunofluorescence analysis of HGC‐27 transfected with si‐circ or co‐transfected anti‐miR upon 5‐FU (6 μM 48 h) and/or CQ (20 μM 24 h) exposure. (H and I) Quantification data of autolysosome and autophagosome in HGC‐27. Scale bar 10 μm. (J) TEM images of HCG‐27‐5‐FU and HGC‐27 with specific treatments. Scale bar = 2 μm or 0.5 μm. Left lower panel: Quantification data of AV counts in HGC‐27‐5FU transfected with si‐circ or co‐transfected anti‐miR upon 5‐FU exposure (30 μM 48 h). Right lower panel: Quantification data of AV counts in HGC‐27 transfected with si‐circ or co‐transfected anti‐miR upon 5‐FU (6 μM 48 h) and/or CQ (20 μM 24 h) exposure. The number of AV of 15 cells was counted in each section. (Graph represents mean ± SD; *p < .05, **p < .01 and ***p < .001) [file CTM2-12-e708-s006.pdf]

**A**

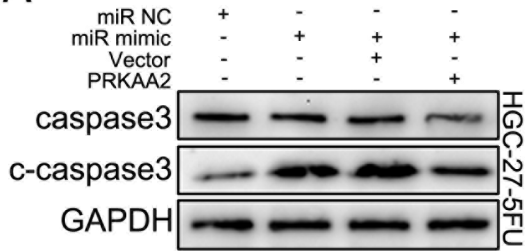

**B**

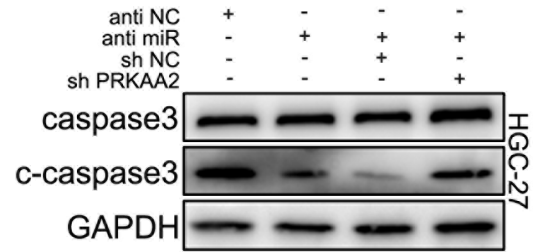

**C**

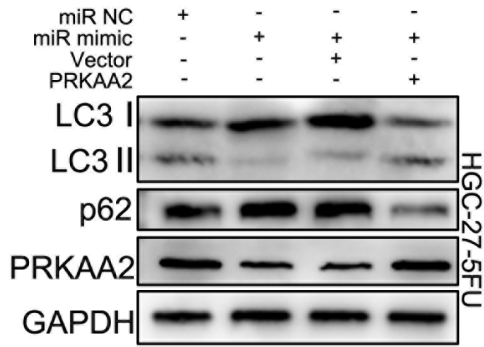

**D**

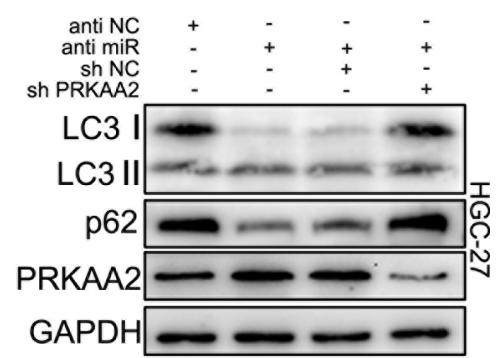

**E**

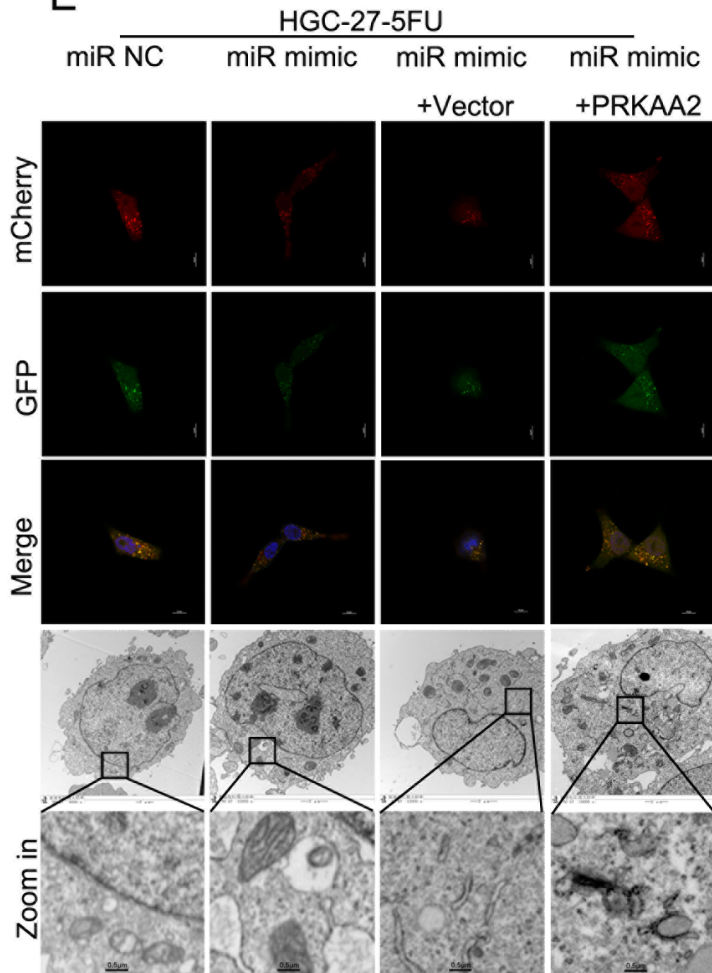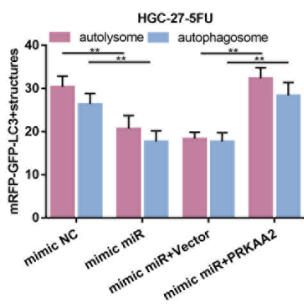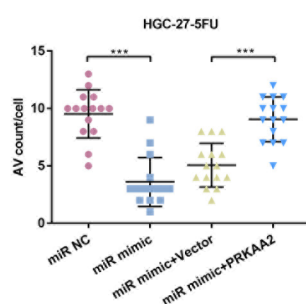

**F**

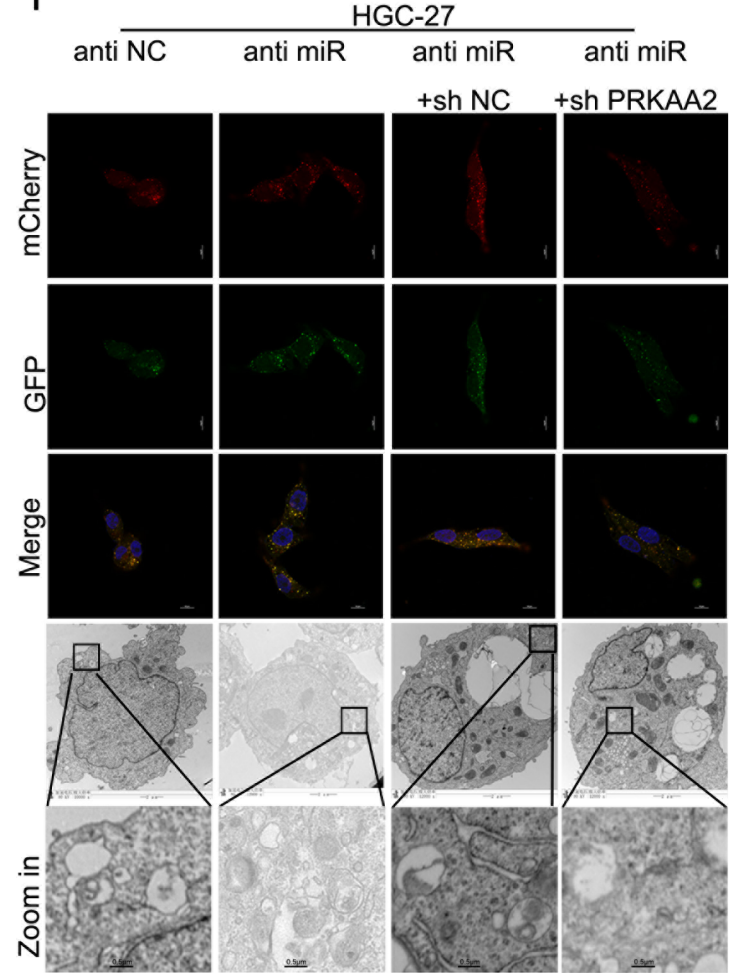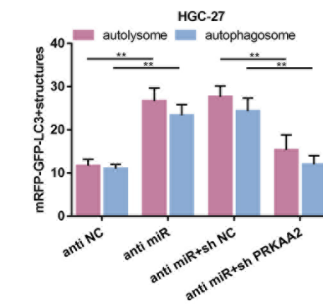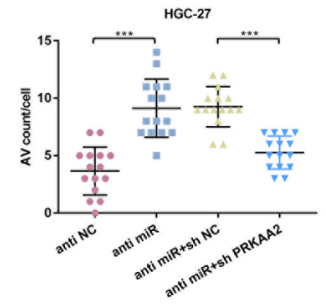

Supplement: Supplementary file 5 — Figure S4 (A and C) Western blot analysis of caspase3, c‐caspase 3, LC3 and p62 in HGC‐27‐5FU transfected with miR mimic or co‐transfected with PRKAA2 overexpression vector upon 5‐FU exposure (30 μM 48 h). (B and D) Western blot analysis of caspase3, c‐caspase 3, LC3 and p62 in HGC‐27 transfected with anti‐miR or co‐transfected with sh‐PRKAA2 in HGC‐27 upon 5‐FU exposure (6 μM 48 h). (E) Immunofluorescence analysis and TEM images of HGC‐27‐5FU transfected with miR mimic or co‐transfected PRKAA2 overexpression vector upon 5‐FU exposure (30 μM 48 h). Scale bar 10 μm. Left lower panel: Quantification data of autolysosome and autophagosome in HGC‐27‐5FU. Right lower panel: Quantification data of AV counts in HGC‐27‐5FU. (F) Immunofluorescence analysis (scale bar 10 μm) and TEM images (scale bar = 2 μm or 0.5 μm) of HGC‐27 transfected with anti‐miR or co‐transfected with sh‐PRKA A2 upon 5‐FU exposure (6 μM 48 h). Left lower panel: Quantification data of autolysosome and autophagosome in HGC‐27. Right lower panel: Quantification data of AV counts in HGC‐27. The number of AV of 15 cells was counted in each section. (Graph represents mean ± SD; *p < .05, **p < .01 and ***p < .001) [file CTM2-12-e708-s001.pdf]

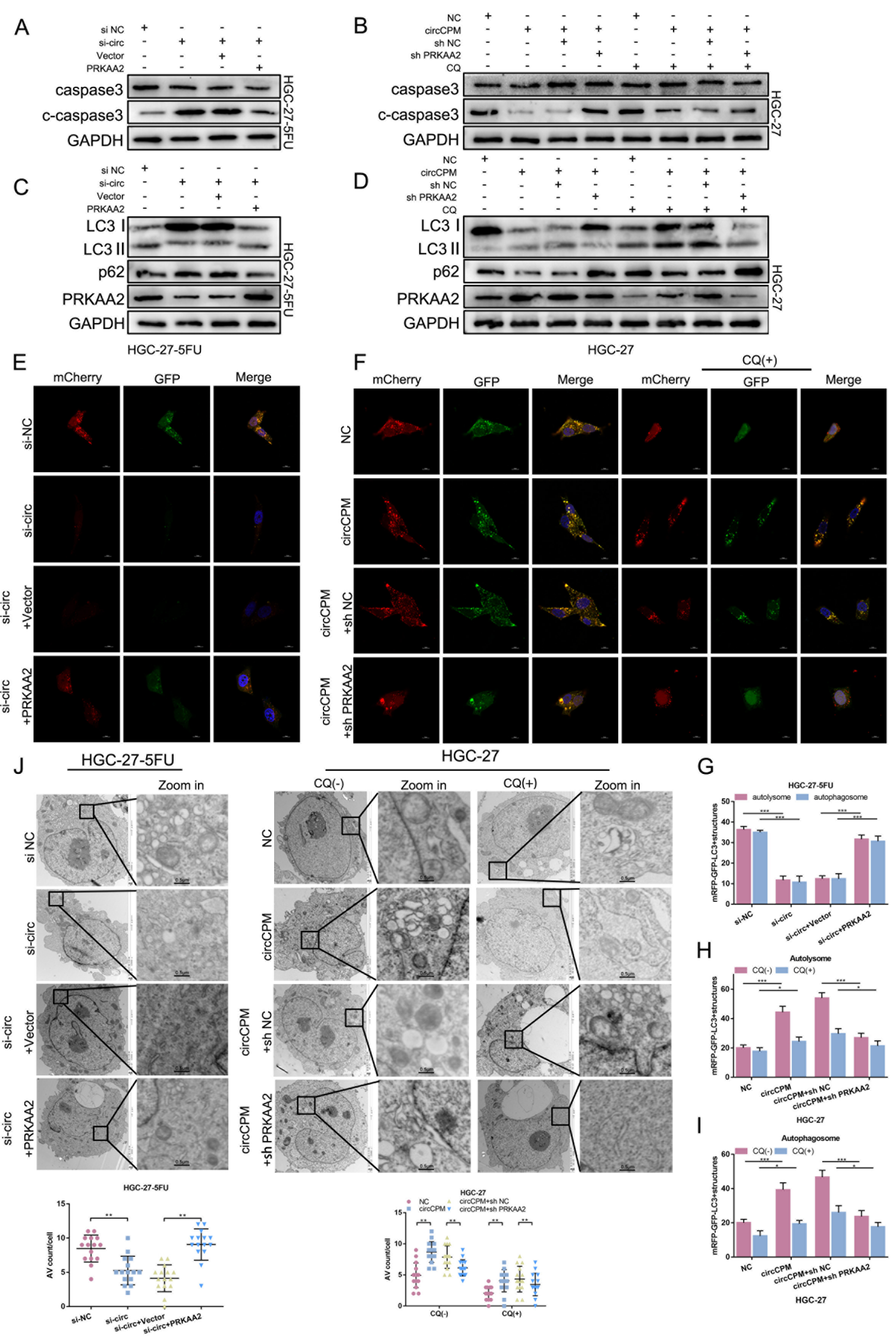

Supplement: Supplementary file 6 — Figure S5 (A and C) Western blot analysis of caspase3, c‐caspase 3, LC3 and p62 in HGC‐27‐5FU transfected with si‐circ or co‐transfected with PRKAA2 overexpression vector upon 5‐FU (30 μM 48 h). (B and D) Western blot analysis of caspase3, c‐caspase 3, LC3 and p62 in HGC‐27 transfected with circCPM overexpression vector or co‐transfected with sh‐PRKAA2 upon 5‐FU (6 μM 48 h) and/or CQ (20 μM 24 h) exposure. (E and G) Immunofluorescence analysis of HGC‐27‐5FU transfected with si‐circ or co‐transfected with PRKAA2 overexpression vector upon 5‐FU (30 μM 48 h). Scale bar 10 μm. (G) quantification data of autolysosome and autophagosome. (F, H and I) Immunofluorescence analysis of HGC‐27 transfected with circCPM overexpression vector or co‐transfected with sh‐PRKAA2 upon 5‐FU (6 μM 48 h) and/or CQ (20 μM 24 h) exposure. Scale bar 10 μm. (H and I) Quantification data of autolysosome and autophagosome. (J) TEM images of HCG‐27‐5‐FU and HGC‐27 with specific treatments. Scale bar = 2 μm or 0.5 μm. Left lower panel: Quantification data of AV counts in HGC‐27‐5FU transfected with si‐circ or co‐transfected with PRKAA2 overexpression vector upon 5‐FU (30 μM 48 h). Right lower panel: Quantification data of AV counts in HGC‐27 transfected with circCPM overexpression vector or co‐transfected with sh‐PRKAA2 upon 5‐FU (6 μM 48 h) and/or CQ (20 μM 24 h) exposure. (Graph represents mean ± SD; *p < .05, **p < .01 and ***p < .001) [file CTM2-12-e708-s005.pdf]
